# Double activation and low IL-2 manufacturing protocol increase expansion of healthy and patient-derived CAR-Tregs

**DOI:** 10.1016/j.omta.2026.201808

**Published:** 2026-07-09

**Authors:** Alessia Ugolini, Anna Miceli, Clara Bercher-Brayer, Susanna Cesarano, Barbara Camisa, Pierluigi Carulli, Paolo Monti, Giuseppe Alvise Ramirez, Elisa Bonomi, Laura Rudilosso, Lorenzo Dagna, Daniela Cesana, Raffaella Greco, Fabio Ciceri, Chiara Bonini, Matteo Doglio

**Affiliations:** 1 Experimental Haematology Unit, Division of Immunology, Transplantation and Infectious Diseases (DITID), IRCCS San Raffaele Scientific Institute, 20132 Milan, Italy; 2 Vita-Salute San Raffaele University, 20132 Milan, Italy; 3 San Raffaele Diabetes Research Institute, IRCCS San Raffaele Hospital, 20132 Milan, Italy; 4 Immunology, Rheumatology, Allergology and Rare Disease Unit, IRCCS San Raffaele Hospital, 20132 Milan, Italy; 5 San Raffaele Telethon Institute for Gene Therapy, IRCCS San Raffaele Scientific Institute, 20132 Milan, Italy; 6 Hematology and Bone Marrow Transplant Unit, IRCCS San Raffaele Hospital, 20132 Milan, Italy

**Keywords:** chimeric antigen receptors, CARs, regulatory T cells, Tregs, CAR-Tregs, manufacturing, engineering, expansion protocols, restimulation, adoptive cell therapy

## Abstract

Chimeric antigen receptor (CAR)-engineered regulatory T cells (CAR-Tregs) are a promising approach to restore immune tolerance in autoimmune diseases (ADs). However, the low frequency of circulating Tregs, especially in patients with autoimmunity, might limit their applicability as a clinical approach. Thus, an optimal protocol for *in vitro* expansion of CAR-Tregs would be highly beneficial. We identified three major variables that could affect Treg expansion: the intensity of TCR triggering to activate the cells, the concentration of IL-2, and the number of activation cycles. We combined these variables to compare different CAR-Treg expansion protocols, starting from CD4+CD25+ Tregs transduced with FOXP3 gene and cultured in IL-2 and rapamycin, and we compared them with our standard approach. Two rounds of Treg activation, at a bead-to-cell ratio of 1:1 promoted the optimal expansion of CAR-Tregs, without affecting their phenotype or suppressive function. Dual-activated CAR-Tregs retained their favorable safety profile and reduced the accumulation of activated conventional T cells in organs in a model of xeno-GvHD. When applied to patient-derived Tregs, the dual-activation protocol promoted a robust and higher expansion compared with our standard strategy, while preserving their functionality.

## Graphical abstract

**Figure:**
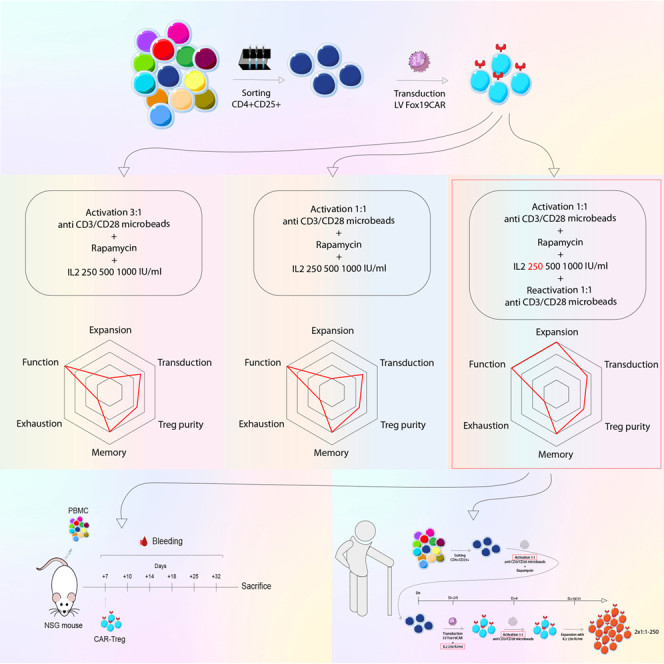

## Teaser

Doglio, Bonini, and colleagues in this original paper, compare different expansion protocols for chimeric antigen receptor-engineered regulatory T cells. Combining the activation intensity, different IL-2-concentrations, and the number of activations, they found that two rounds of activation promote cell expansion without affecting the cell phenotype and function.

## Introduction

Regulatory T cells engineered with chimeric antigen receptors (CAR-Tregs) are a promising new advanced therapy medicinal product for the treatment of autoimmune diseases (ADs) and for the prevention of rejection in solid organ transplantation.^1^^,^^2^^,^^3^ Several protocols for Treg expansion have been proposed. Different isolation strategies, the number of activation cycles with anti-CD3/CD28 microbeads, the bead-to-T cell ratios, the use of additional compounds such as rapamycin, and the IL-2 concentration are the most significant differences between the various approaches.^4^^,^^5^^,^^6^ However, a comparative analysis aimed at identifying the optimal manufacturing procedure is still lacking. Due to the low frequency of Tregs in the peripheral blood, robust protocols for their expansion are required to ensure the clinical applicability of this approach. This is particularly crucial for autologous products, considering the reduced number of circulating Tregs and their dysfunctional state in patients with autoimmunity, especially if exposed to immunosuppressive treatments.^7^ All these elements might significantly affect the feasibility of CAR-Treg-based strategies.

In 2006, Battaglia et al. proposed the use of anti-CD3/CD28 microbeads and rapamycin to selectively expand polyclonal Tregs from patients with type 1 diabetes (T1D).^8^ By modifying this protocol to include the transduction with a lentiviral vector (LV) encoding for a CAR and for FOXP3, in 2024, we proposed an efficient platform for stable CAR-Treg generation starting from CD4+CD25+ cells, which represents a good balance in terms of cell number and purity.^9^

With the ultimate aim of clinical translation of CAR-Treg cellular products, here, we combined and compared different biological variables of the CAR-Treg manufacturing procedure to achieve greater cell expansion and yield of functional and pure engineered Tregs. We show that two rounds of Treg activation are crucial to promote the proper expansion of CD4+CD25+-derived CAR-Tregs without affecting their functionality.

## Results

### Consistent CAR-Treg expansion, phenotype, and function across variable IL-2 levels and activation conditions

We previously established a protocol for stable CAR-Treg generation, based on the co-transduction of a CAR construct and the *FOXP3* gene followed by culturing in rapamycin and 500 IU/mL IL-2.^9^ Although this approach achieved up to 30-fold CAR-Treg expansion in healthy donors, the intrinsic dysfunction of patient-derived Tregs may limit its clinical translatability.^7^ To optimize *ex vivo* manipulation, we investigated three critical factors identified in the literature: IL-2 concentration, anti-CD3/CD28 bead-to-T cell ratio, and stimulation timing.^10^ Our goal was to identify the most robust protocol for CAR-Tregs transduced with *FOXP3* and cultured with rapamycin and IL-2.

**Figure 1:**
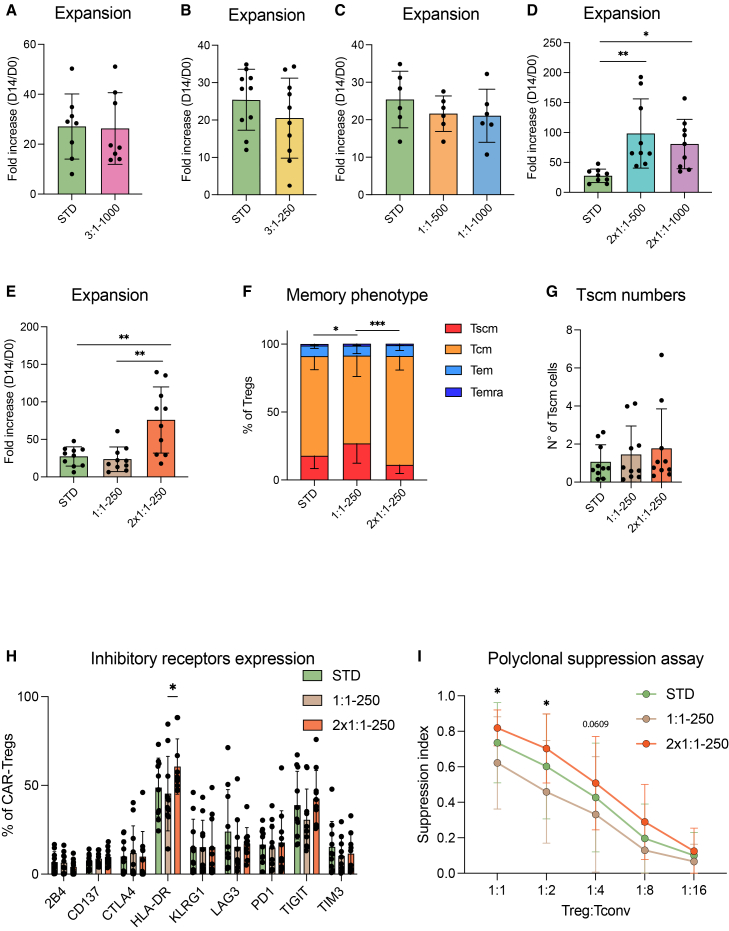
Screening of CAR-Treg expansion protocols (A–E) Expansion rate of CAR-Tregs cultured with different expansion protocols: the standard protocol (STD) was based on 1 round of Treg activation with CD3/CD28 beads at a 3:1 beads-to-T cell ratio and IL-2 at 500 IU/mL. New protocols used 3:1 or 1:1 beads-to-T cell ratios with IL-2 at 250, 500, or 1,000 IU/mL. Protocols involving 2 rounds of Treg activation at 1:1 beads-to-T cell ratio (2× 1:1) with IL-2 at 250, 500 or 1,000 IU/mL were also tested (legend in Figure S1). Fold increase was calculated dividing the number of cells obtained at day +14 with the one at day 0. Two-way ANOVA with Tukey correction for multiple comparison ∗*p* < 0.0127, 2× 1:1–1,000 vs. STD; ∗∗*p* < 0.0014, 2× 1:1–500 vs. STD; ∗∗*p* < 0.0051, 2× 1:1–250 vs. STD; ∗∗*p* < 0.0028, 2× 1:1–250 vs. 1:1–250. (F) Percentage of memory subsets detected by flow cytometry at day +21, identified as follows: CD62L+CD45RA+ memory stem T cells (Tscm), CD62L+CD45RA− central memory T cells (Tcm), CD62L−CD45RA− effector memory T cells (Tem), and CD62L−CD45RA+ effector memory T cells CD45RA+ (Temra). Two-way ANOVA with Tukey correction for multiple comparison ∗*p* < 0.0496, ∗∗∗*p* < 0.0001. *n* = 10. (G) Absolute number of Tscm cells in the different expansion protocols. (H) Percentage of CAR-Treg (CD3+CD4+CD25+FOXP3+CAR+) cells expressing activation and exhaustion markers detected by flow cytometry after 21 days of culture. Two-way ANOVA with Tukey correction for multiple comparison ∗*p* < 0.0389. *n* = 10. (I) Polyclonal suppressive capacities of CAR-Tregs activated at different Treg:Tconv ratios. Results are expressed in terms of suppression index. One-way ANOVA with Tukey correction for multiple comparison ∗*p* < 0.0432 for 1:1, ∗*p* < 0.0263 for 1:2. *n* = 9. All data are represented as mean ± SD. (A) *n* = 8; (B) *n* = 10; (C) *n* = 6; (D) *n* = 9; (E–I) *n* = 10 biological replicates.

Given the high IL-2 dependency,^11^ we assessed the role of different IL-2 concentrations on CAR-Treg expansion, phenotype, and functionality. CD4+CD25+ Tregs isolated from healthy donors were activated with anti-CD3/CD28 beads (3:1 bead-to-T cell ratio) in the presence of rapamycin. After 2 days, the cells were transduced with a second-generation anti-CD19 CAR-FOXP3 LV (Fox19CAR LV).^9^ Starting from day +3, cultures were supplemented with either 1,000 (3:1–1,000) or 250 (3:1–250) IU/mL IL-2 every 2–3 days. As control, Tregs cultured with our standard IL-2 (500 IU/mL, STD) were included (Figures S1A and S1C; Table 1). After 14 days, no differences in expansion rate were observed across IL-2 concentrations (Figures 1A and 1B).

**Table 1:** Schematic representation of the different expansion protocols

IL-2 concentration (IU/mL)			Beads-to-cell ratio		Additional activation		Protocol name
250	500	1,000	1:1	3:1	Yes	No	
X	–	–	–	X	–	X	3:1–250
–	X	–	–	X	–	X	STD
–	–	X	–	X	–	X	3:1–1,000
X	–	–	X	–	–	X	1:1–250
–	X	–	X	–	–	X	1:1–500
–	–	X	X	–	–	X	1:1–1,000
X	–	–	X	–	X	–	2× 1:1–250/DS
–	X	–	X	–	X	–	2× 1:1–500
–	–	X	X	–	X	–	2× 1:1–1,000

We then investigated whether the TCR activation strength influences the Treg yield.^4^^,^^12^ Although anti-CD3/CD28 beads are standard for activation, the optimal bead-to-Treg ratio remains poorly defined. We thus explored the impact of a reduced anti-CD3/CD28 bead-to-T cell ratio (1:1 bead-to-T cell ratio) on the expansion and functionality of healthy donors’ CAR-Tregs, cultured in the presence of either 500 IU/mL (1:1–500) or 1,000 (1:1–1,000) IU/mL of IL-2, compared with our standard approach (Figures S1A, S1E, and S1F; Table 1). After 14 days of culture, we found that a reduced bead-to-T cell ratio did not modify CAR-Treg expansion independently from the IL-2 concentration.

A comprehensive product characterization was performed at day 21, revealing no difference across all cellular products in terms of transduction efficiency and purity compared with our standard approach (Figures S2A–S2I). The memory phenotype was similarly unaffected, with central memory T cells ([Tcm], CD45RA−CD62L+) representing the most abundant subset, followed by memory stem T cells ([Tscm], CD45RA+ CD62L+). Moreover, flow cytometry analysis of nine activation and exhaustion markers (including LAG3, PD1, TIGIT, and TIM3) showed no significant upregulation compared with our standard product (Figures S3A, S3C, and S3E). Finally, all the cellular products retained their suppressive abilities against autologous conventional T cells upon polyclonal stimulation (Figures S3B, S3D, and S3F).

Taken together, these findings indicate that, within a single-activation framework, neither IL-2 concentration nor bead-to-T cell ratio represents major determinants of CAR-Treg expansion or function. This observation allowed us to isolate the contribution of the activation strategy itself—specifically, the number of stimulation rounds—as the primary variable to be investigated in subsequent experiments.

### Double activation with a low bead-to-T cell ratio promotes the expansion of highly suppressive CAR-Tregs

Several CAR-Treg expansion protocols described in the literature involve an activation phase consisting of two distinct stimulation rounds. To evaluate whether this approach enhances cell expansion, we exposed Tregs to two rounds of activation (day 0 and +9) using a 1:1 anti-CD3/CD28 bead-to-T cell ratio.^13^ This was tested at two different IL-2 concentrations, 500 IU/mL (2× 1:1–500) or 1,000 IU/mL (2× 1:1–1,000) and compared against our standard protocol (STD) (Figures S1H–S1I; Table 1). By day 14, double-stimulated CAR-Tregs showed a significantly higher expansion rate than the standard approach, regardless of the IL-2 concentration (mean [SD]: 2× 1:1–500, 98.30 [57.78]; 2× 1:1–1,000, 80.73 [41.24]; STD, 27.64 [11.23]; STD vs. 2× 1:1–500, *p* value <0.0014; STD vs. 2× 1:1–1,000, *p* value <0.0127) (Figure 1D).

To confirm the robustness to IL-2 variations, we evaluated the CAR-Treg expansion at 250 IU/mL IL-2 and stimulated with one (1:1–250) or two rounds (2× 1:1–250) of activation with anti-CD3/CD28 beads at a 1:1 bead-to-T cell ratio. (Figures S1A, S1D, and S1G; Table 1). At day +14, double-activated CAR-Tregs exhibited significantly greater expansion than both 1:1–250 and STD controls (mean [SD]: STD, 27.25 [12.83]; 1:1–250, 23.57 [16.41]; 2× 1:1–250, 75.84 [44.14]; *p* value <0.0028, 2× 1:1–250 vs. 1:1–250; *p* value <0.0051, 2× 1:1–250 vs. STD) with a mean value comparable to the other double activation protocols tested (Figure 1E).

We next characterized the cellular products in terms of phenotype and function. Compared with the controls, the two-step stimulation did not compromise the transduction efficiency and purity at day +21, independently from the IL-2 concentration (Figures S4A–S4D). Regarding the memory phenotype, most groups showed comparable results. Single-stimulated CAR-Tregs maintained in 250 IU/mL IL-2 exhibited a higher Tscm frequency compared with double-stimulated cells in the same IL-2 concentration and compared with the standard protocol. However, when accounting for the total cellular expansion, the absolute number of Tscm cells remained comparable between the two protocols, suggesting that the increased yield compensates for the lower percentage (Tscm 1:1–250, 26.54 [14.53]; STD, 17.69 [9.32]; 2× 1:1–250, 10.73 [6.30]; *p* value <0.0001, 1:1–250 vs. 2× 1:1–250; *p* value <0.0496, 1:1–250 vs. STD) (Figures 1F and 1G; Figure S4E).

Flow cytometry analysis showed a higher frequency of activated HLA-DR+ cells in the 2× 1:1–250 group compared with the single-stimulated condition (mean [SD]: 2× 1:1–250, 20.94 [18.44]; 1:1–250, 17.66 (12.50); *p* value <0.0389) (Figure 1H), while for the other protocols it was unremarkable (Figure S4F). Functionally, double-stimulated CAR-Tregs suppressed T cell proliferation upon polyclonal stimulation comparably to the standard (Figure S4H). Notably, within the 250 IU/mL IL-2 conditions, double-stimulated CAR-Tregs significantly outperformed single-stimulated ones (*p* value <0.0432 and *p* value <0.0263 in 1:1 and 1:2 Treg:PBMC [peripheral blood mononuclear cell] ratio) (Figure 1I).

Collectively, these results demonstrate a clear proliferative advantage of the dual-activation strategy at a 1:1 bead-to-T cell ratio over single-activation protocols without sacrificing functional potency and regardless of the IL-2 concentration in the culture.

### The dual activation strategy preserves antigen-specific suppressive capacities without reprogramming into CD4+CD25− Tconvs

Current CAR-T cell manufacturing trends favor “fast” protocols to enhance feasibility and reduce costs.^14^ However, given the low frequency and the low expansion capacity of circulating Tregs, we prioritized protocol robustness and product stability over manufacturing speed and simplicity. To validate this approach, we assessed two key features of the CAR-Treg product: antigen-specific suppressive capacity and the stability of both CAR-Tregs and induced CAR-Tregs (CAR-iTregs)—the latter potentially arising from a minor CD4+CD25− conventional T cell fraction in the starting material.

**Figure 2:**
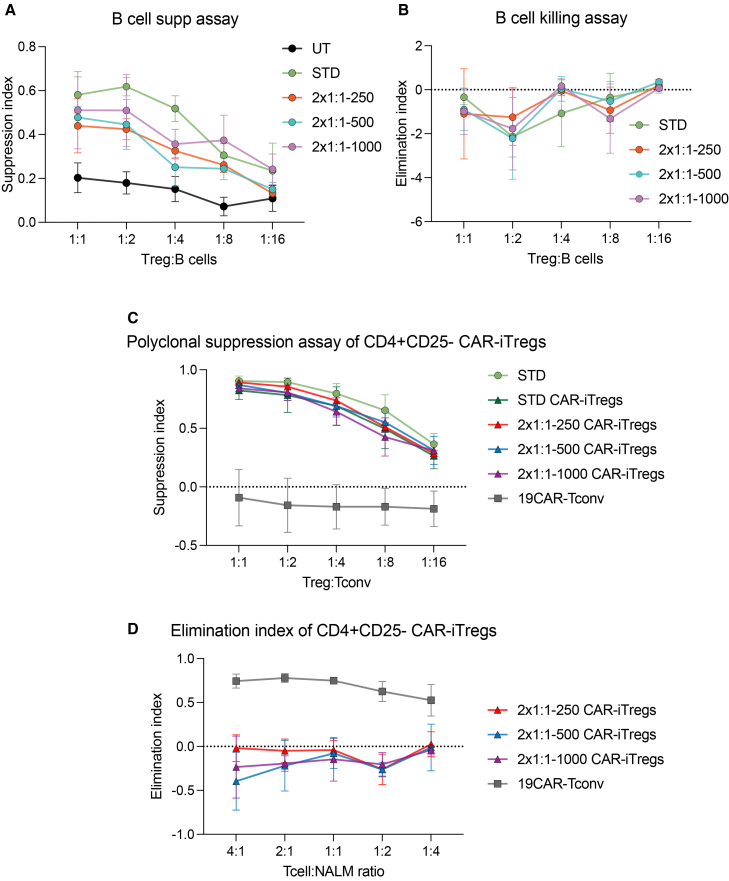
Functional characterization of restimulated products (2× 1:1–250, 2× 1:1–500, and 2× 1:1–1,000) and safety validation (A) Comparison of 2× 1:1–250, 2× 1:1–500, and 2× 1:1–1,000 CAR-Treg suppressive abilities with STD CAR-Tregs and untransduced Tregs (UT) upon antigen-specific activation mediated by autologous B cells. Results are expressed in terms of suppression index. (B) Killing capacities of CAR-Treg populations assessed after 3-day coculture in the presence of B cells as targets. Results are expressed as elimination index over untransduced Tregs (UT). (C) Polyclonal suppressive capacities of CD4+CD25− CAR-iTregs at different Treg-to-Tconv ratios. CD4+CD25+ CAR-Tregs STD and CD4+CD25− CAR-Tconv were used as controls. Results are expressed in terms of suppression index. (D) Killing capacities of CD4+CD25− CAR-iTreg populations assessed after 3-day coculture in the presence of B cells as target. Results are expressed as elimination index over CD4+CD25+ CAR-Tregs STD. All data are represented as mean ± SD. (A–D) *n* = 3 biological replicates.

Using an anti-CD19 CAR, we assessed the proliferation of CD19+ B cells from healthy donors upon stimulation with 2× 1:1–250, 2× 1:1–500, and 2× 1:1–1,000 CAR-Tregs and we compared it with that of B cells cultured with our cells engineered with our standard protocol (STD) or with polyclonal Tregs cultured with our standard protocol (UT). Dual-activated CAR-Tregs were as effective as STD cells in suppressing B cell proliferation (Figure 2A). To exclude the acquisition of cytotoxic activity, we assessed CAR-Treg-mediated killing of B cells. Similar to the untransduced cells, engineered Tregs did not kill target cells (Figure 2B).

Although CD4+CD25+ sorting yields a highly enriched Treg population, a minor CD4+CD25− conventional T cell fraction is frequently detected in the starting material. It is, therefore, critical to ensure that the manufacturing process is robust regardless of the CD4+CD25− contaminant fraction and that this population does not acquire undesired functional properties. We previously showed that the addition of the *FOXP3* gene can reprogram CD4+CD25− T cells into a suppressive phenotype when cultured in the presence of IL-2 and rapamycin.^9^ Given that the additional stimulation round represents a new variable not included in our previously validated protocol, we specifically investigated whether this modification alters the reprogramming trajectory of the CD4+CD25− fraction (CAR-iTreg).

As a proof of concept, we isolated CD4+CD25− cells from healthy donors and we stimulated them with two rounds of anti-CD3/CD28 beads in a 1:1 bead-to-T cell ratio. CAR-iTregs were cultured with rapamycin at different IL-2 concentrations (250, 500 or 1,000 IU/mL) and compared with CAR-Tregs and CAR-iTregs obtained with our standard protocol (STD). In addition, a fraction of CD4+CD25− cells were cultured in the presence of IL-7 and IL-15 and transduced with a CAR19.28z construct devoid of the *FOXP3* gene, in the absence of rapamycin to obtain conventional cytotoxic CAR-T cells (CAR-Tconvs)^15^^,^^16^ (Figure S5A).

Unlike CAR-Tconvs, all dual-activated CAR-iTregs suppressed PBMC proliferation upon polyclonal stimulation as effectively as STD CAR-Tregs or CAR-iTregs cultured with the standard protocol (Figure 2C). To further investigate their cytotoxicity, we cultured CAR-iTregs with Nalm-6, a CD19+ tumor cell line. Compared with the STD-engineered Tregs, CD4+CD25− T cell-derived CAR-iTregs did not exhibit cytotoxic activity against CD19+ targets, even at the most challenging T cell: target ratios. In contrast, CAR-Tconvs eliminated all tumor cells at all tested conditions (Figure 2D).

### Double-activated CAR-Tregs retain broad suppressive function, phenotypic stability, and a favorable genotoxicity profile

Dual-activation protocol enables the generation of fully functional, stable CAR-Tregs with enhanced expansion. Considering that high level of IL-2 may negatively affect Treg suppressive function^12^^,^^17^ and that minimizing cytokine use is also relevant for clinical-grade manufacturing costs, we selected the 250 IU/mL IL-2 dual-activation protocol (DS) for subsequent in-depth characterization.

**Figure 3:**
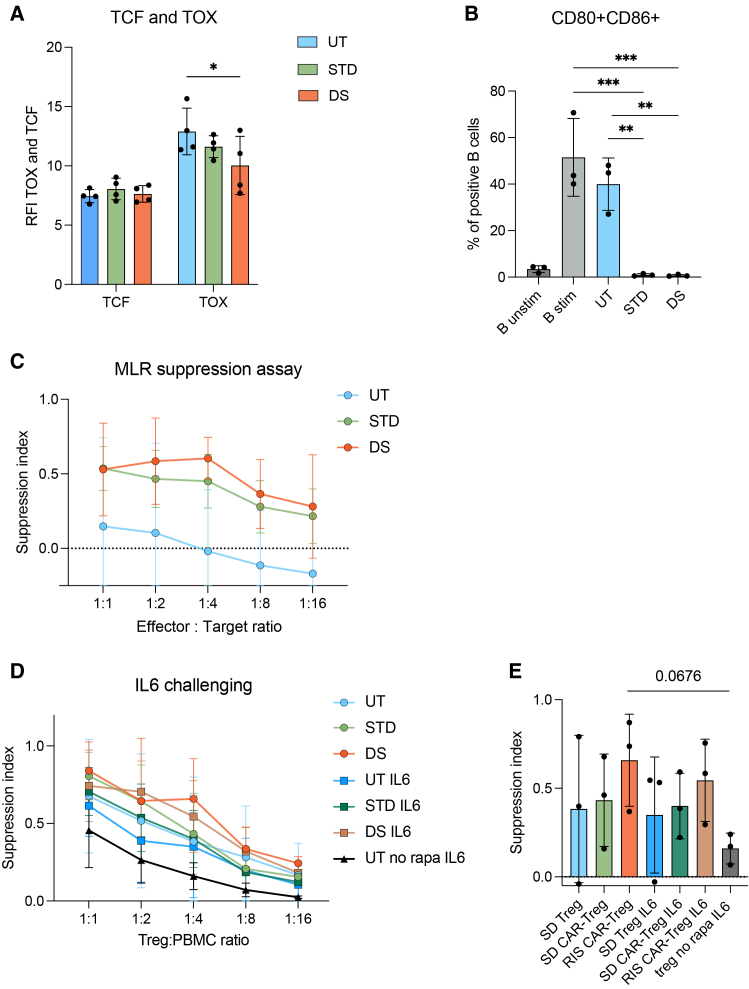
Double-activated CAR-Tregs retain broad suppressive function and phenotypic stability (A) RFI of TCF1/7 and TOX expression of untransduced Tregs (UT), standard protocol CAR-Tregs (STD), and double-activated CAR-Tregs (DS) over FMO. Two-way ANOVA with Tukey correction for multiple comparison: ∗*p* < 0.0290. (B) Percentage of B cells co-expressing the CD80 and CD86 markers after 72-h coculture with Tregs or left alone. Two-way ANOVA with Tukey correction for multiple comparison ∗∗∗*p* <0.001, UT vs. STD; ∗∗*p* <0.0025, UT vs. DS; ∗∗ *p* <0.0024. (C) Suppressive abilities of Tregs on autologous PBMCs upon allogeneic stimulation. Results are expressed in terms of suppression index. (D and E) Polyclonal suppressive abilities of CAR-Tregs cultured in the presence or absence of IL-6 in comparison with polyclonal Tregs cultured without rapamycin (UT no rapa IL-6). Results are expressed in terms of suppression index. All data are represented as mean ± SD. (A-E) *n* = 3 biological replicates.

Repeated stimulation may induce CAR-Treg exhaustion, potentially impairing their function.^17^ As shown in Figure 1H, flow cytometry analysis did not reveal upregulation of exhaustion markers on dual-activated CAR-Tregs compared with our standard cellular product. We further assessed this by measuring two transcription factors associated with exhaustion, T cell factor (TCF) and thymocyte selection-associated HMG-box (TOX),^18^ in our cellular products. TCF and TOX expressions were similar in dual-activated and standard CAR-Tregs at day +21. Notably, compared with untransduced Tregs processed with the standard protocol, dual-activated cells showed a reduced TOX expression (*p* value <0.0290), while TCF was similar (Figure 3A).

Anti-CD19 CAR-Tregs have been shown to exert strong control over the inflammatory response at the tissue level by acting on multiple immune cells.^6^^,^^9^ We thus further evaluated the suppressive capacity of dual-activated CAR-Tregs. First, we assessed their capacity to control B cell antigen-presenting cell activity. When exposed to CD19-specific CAR-Tregs, independently from the manufacturing protocol used (STD or dual activation [DS]), stimulated B cells downregulated CD80 and CD86, two fundamental co-stimulatory molecules,^19^ while no effect was detected when B cells were exposed to untransduced Tregs (*p* value <0.0001) (Figure 3B; Figures S6A–S6C). We then confirmed CAR-Treg-suppressive capacities against T cells, with both CAR-Treg products efficiently controlling T cell proliferation upon allogeneic stimulation in a mixed lymphocyte reaction, compared with the untransduced Tregs (Figure 3C).

To further investigate safety, we evaluated the phenotypic stability of engineered cells in a pro-inflammatory environment. In contrast to untransduced Tregs, CAR-Tregs displayed similar suppressive capacities against PBMCs upon polyclonal stimulation in the presence or absence of IL-6 *in vitro* (Figures 3D and 3E). To evaluate the combined safety of lentiviral engineering and the dual-activation expansion protocol, we investigated whether this manufacturing strategy could promote the preferential expansion of only a subset of transduced clones. Vector copy number was comparable between standard and dual-activated CAR-Treg products. A more detailed quantitative analysis of vector integration sites, performed by sonication-based linker-mediated PCR, showed a similar overall integration burden in the two conditions and preserved clonal diversity, as measured by the Shannon diversity index. These data indicate that dual activation does not induce clonal skewing, but rather supports a homogeneous polyclonal expansion of transduced CAR-Tregs. This was further supported by the Gene Ontology (GO) enrichment analysis, which did not identify enrichment of specific gene sets and was comparable between the two CAR-Treg populations and with untransduced Tregs (Figures S7A–S7E).

Taken together, these data confirm the functionality of dual-activated CAR-Tregs and highlight their broad suppressive capacities against B and T cells. In addition, we showed that double stimulation does not induce an exhausted Treg phenotype, thus preserving their functionality. Finally, we confirmed the safety profile of CAR-Treg by demonstrating their stability in a pro-inflammatory environment and the absence of genotoxicity signals.

### Dual-activated CAR-Tregs control xeno-Graft-versus-Host Disease (GvHD) in a humanized mouse model

**Figure 4:**
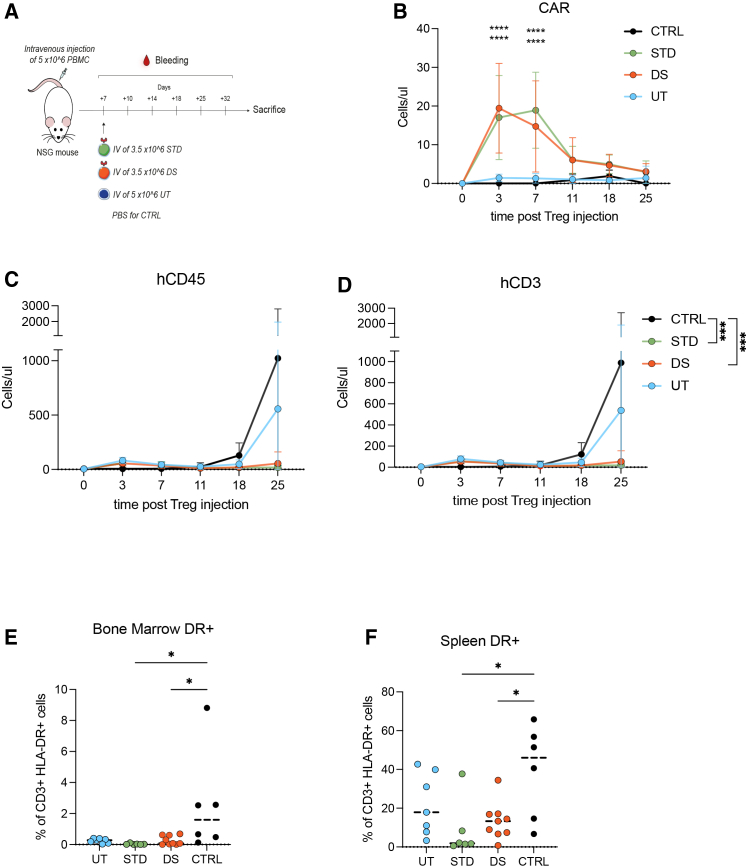
Dual-activated CAR-Treg control xeno-GvHD in a humanized mouse model (A) Schematic representation of the *in vivo* experiment. (B) Count of CAR+ cells in the peripheral blood at different time points post-injection. Results are expressed in cells per microliter. Two-way ANOVA with Tukey correction for multiple comparison ∗∗∗∗*p* <0.0001. (C and D) Count of CD45+ and CD3+ cells in the peripheral blood at different time points post-injection. Results are expressed in cells per microliter. Two-way ANOVA with Tukey correction for multiple comparison CTRL vs. DS, ∗∗∗*p* < 0.0003; CTRL vs. STD, ∗∗∗*p* < 0.0007. (E and F) Percentage of CD3+HLA-DR+ cells over total CD3+ cells in the bone marrow and spleen of treated mice. Two-way ANOVA with Tukey correction for multiple comparison. Bone marrow CTRL vs. STD, ∗*p* < 0.0375; CTRL vs. DS, ∗*p* <0.0394; spleen CTRL vs. STD, ∗*p* <0.0141; CTRL vs. DS, ∗*p* <0.0243. (B–F) UT *n* = 7; STD *n* = 6; DS *n* = 9; CTRL *n* = 6.

CAR-Tregs have been shown to broadly control inflammatory responses at the tissue level.^9^^,^^20^ To confirm preservation of the suppressive capacity in dual-activated CAR-Tregs, compared with standard CAR-Tregs, we used a xeno-GvHD model in NSG mice.^6^ Adult mice were injected with 5 × 10^6^ human PBMCs to induce xeno-GvHD (28 total mice). After 7 days, animals were randomized and treated intravenously with 3.5 × 10^6^ autologous standard (STD; 6 mice), dual-activated CAR-Tregs (DS; 9 mice), or autologous untransduced Tregs generated with the standard protocol (UT; 7 mice). As control (CTRL), 6 mice received PBS intravenously (Figure 4A).

Following treatment, circulating human immune cells were monitored by flow cytometry. Both CAR-Treg products expanded significantly more than untransduced Tregs during the first week after injection and then progressively contracted (*p* value <0.0001) (Figure 4B). Starting from day +18, we observed a significant increase of hCD45+ and hCD3+ cells in the peripheral blood of PBS-treated animals compared with the CAR-Treg-treated groups (CTRL vs. DS, *p* value <0.0003; CTRL vs. STD, *p* value <0.0007) (Figures 4C and 4D).

At day +25, animals were euthanized. We quantified the human cell infiltration in the spleen and bone marrow by flow cytometry. While total CD3+ frequencies were comparable across groups, dual-activated CAR-Tregs significantly reduced the percentage of activated (HLA-DR+) human T cells in the bone marrow and spleen compared with PBS-injected controls, with no difference between the two CAR-Treg products (bone marrow CTRL vs. STD, *p* value <0.0375; CTRL vs. DS, *p* value <0.0394; spleen CTRL vs. STD, *p* value <0.0141; CTRL vs. DS, *p* value <0.0243) (Figures 4E–4J).

These results demonstrate that dual-activated CAR-Tregs retain *in vivo* expansion capacity and the ability to control peripheral T cell expansion at levels comparable to standard CAR-Tregs. Notably, the reduction of activated T cells in target organs suggests effective disease modulation. Taken together, these findings indicate that the dual-activation protocol yields a CAR-Treg product with *in vivo* efficacy comparable to the standard manufacturing process.

### The dual-activation strategy allows superior fully functional patient-derived CAR-Treg expansion

**Figure 5:**
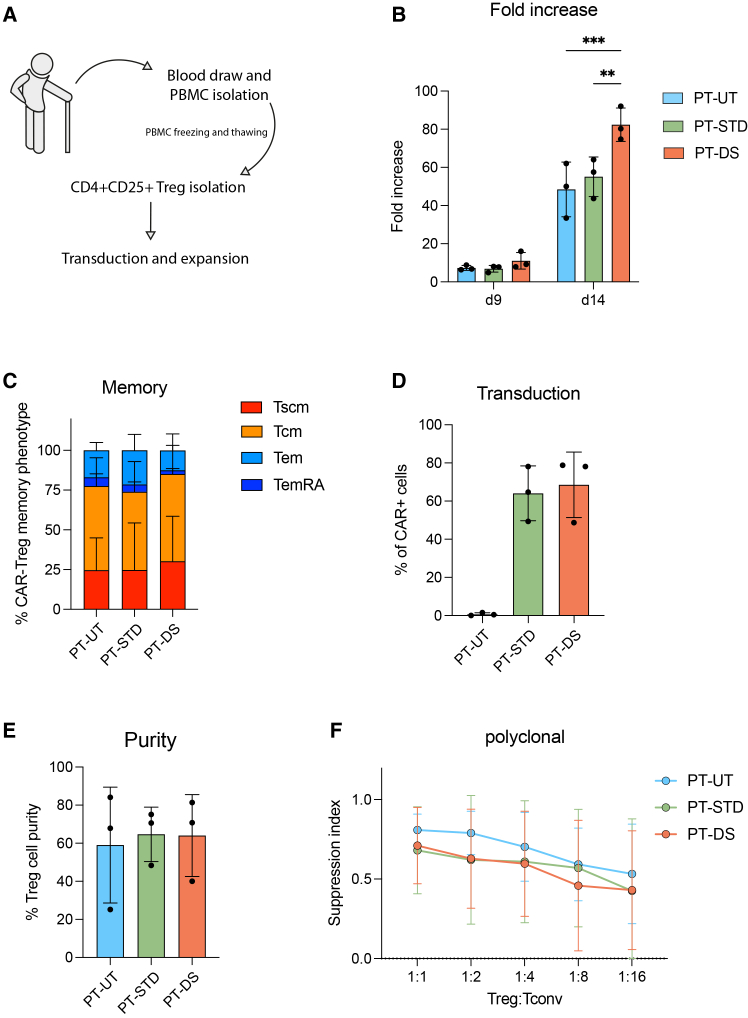
The dual activation strategy allows superior fully functional patient-derived CAR-Treg expansion (A) Schematic representation of patient cells workflow. (B) Expansion rate of untransduced patient-derived Tregs (UT) or CAR-Tregs cultured with the standard protocol (STD) or the double-activation protocol (DS). Fold increase was calculated by dividing the number of cells obtained at day +14 with the one at day 0. Two-way ANOVA with Tukey correction for multiple comparison UT-PT vs. DS-PT, ∗∗∗*p* <0.0008; STD-PT vs. DS-PT, ∗∗*p* <0.0045. (C) Percentage of memory subsets detected by flow cytometry at day +21, identified as follows: CD62L+CD45RA+ memory stem T cells (Tscm), CD62L+CD45RA− central memory T cells (Tcm), CD62L−CD45RA− effector memory T cells (Tem), and CD62L−CD45RA+ effector memory T cells CD45RA+ (Temra). Results are expressed as percentage of CD3+CD4+CD25+CD127−FOXP3+ cells. (D) Transduction efficiency of Fox19CAR LV on Tregs assessed by flow cytometry at day +14. CAR+ cells were identified with CD19 CAR FMC63 idiotype antibody. (E) Percentage of regulatory T cells among T lymphocytes at day +21 defined as CD3+CD4+CD25+CD127−FOXP3+ cells. (F) Polyclonal suppressive capacities of CAR-Tregs at different Treg:Tconv ratios. Results are expressed in terms of the suppression index. All data are represented as mean ± SD. (A–F) *n* = 3 patients.

To verify whether the dual-activation strategy enhances the expansion of Tregs derived from patients with ADs, we isolated CD4+CD25+ Tregs from cryopreserved PBMCs of two patients with biologically naive active rheumatoid arthritis (RA) and one with T1D who had undergone pancreatic islet transplantation. Tregs were expanded using the dual-activation protocol in the presence of 250 IU/mL of IL-2 and rapamycin and transduced with the Fox19CAR LV (PT-DS). For comparison, patient-derived Tregs were expanded using the standard protocol (PT-STD) or maintained as untransduced controls (PT-UT) (Figure 5A).

After 14 days, PT-DS cells showed significantly higher expansion than both PT-STD and PT-UT Tregs (PT-UT vs. PT-DS, *p* value <0.0008; PT-STD vs. PT-DS, *p* value <0.0045) (Figure 5B). Importantly, this increased expansion did not affect transduction efficiency, purity, or memory phenotype (Figures 5C–5E). Furthermore, suppressive capacity against healthy donor-derived PBMCs was comparable across PT-DS, PT-STD, and PT-UT products upon polyclonal stimulation (Figure 5F).

Collectively, these findings show that the optimized protocol confers a proliferative advantage to patient-derived Tregs without compromising functional or phenotypic integrity, consistent with observations in healthy donors.

## Discussion

CAR-Tregs are promising therapeutics strategies for ADs and for the prevention of graft rejection and graft-versus-host disease^21^ in transplantation settings. However, regulatory T cells represent only 5%–10% of CD4+ circulating cells and are a heterogeneous population composed of different subsets.^22^ Different sorting strategies have been developed to isolate Tregs, requiring careful assessment of the balance between cell yield and purity.^23^ Furthermore, several Treg expansion protocols have been reported, although a direct comparison to identify the most promising approach is lacking.^24^^,^^25^^,^^26^ Altogether, all these elements contribute to generate a high heterogeneity of CAR-Treg products, thus hindering the identification of the best approach.

In patients affected by ADs, several publications reported a reduction of circulating Tregs, which also displayed a dysfunctional state.^27^ The use of immunosuppressive drugs further exacerbates this aspect by inducing lymphopenia and dampening T cell function.^28^ For these reasons, more permissive sorting strategies, such as the selection of CD4+CD25+ cells, together with a tight control of contaminant Tconvs might improve the feasibility of autologous CAR-Tregs.

Recently, we proposed a CAR-Treg product generated starting with sorted CD4+CD25+ Tregs, which are transduced with a LV encoding for a CAR and the *FOXP3* transgene, after stimulation with anti-CD3/CD28 beads with a bead-to-T cell ratio of 3:1 and cultured in the presence of rapamycin and IL-2 500 IU/mL. The use of rapamycin, either *ex vivo*^8^ or *in vivo*,^29^ has been shown to expand and stabilize Tregs, by inhibiting the mTOR pathway and counterbalancing the negative effects of TCR triggering on the Treg functional phenotype.^30^

These cells proved effective both *in vitro* and *in vivo* in controlling the inflammatory response. This approach allows a higher starting population than with more refined sorting strategies, a high cell yield at the end of the culture, and an optimal control of the contaminant Tconvs.^9^

In the literature, different bead-to-T cell ratios, rounds of stimulation, and IL-2 concentrations have been reported.^4^^,^^5^^,^^6^ To further improve the CAR-Treg manufacturing, we decided to assess the weight of each of these variables on the expansion, transduction, phenotype, and suppressive capacity of CD4+CD25+ T cell-derived CAR-Tregs transduced with a LV encoding an anti-CD19 CAR and the *FOXP3* transgene and cultured in the presence of rapamycin. The introduction of a second stimulation step at day +9 provided a boost to the healthy donor cell expansion and to the final cell yield at day +14 compared with our standard protocol, without affecting the CAR-Treg suppressiveness. The introduction of the second step of stimulation at day +9 is consistent with protocols reported in the literature.^13^^,^^31^ Given the positive impact of dual activation, future refinements may include optimizing the timing for the second stimulation.

Currently, most of the clinical trials utilizing CAR-T cells and CAR-Tregs rely on autologous products to minimize the risk of allogeneic activation^32^; the need for immunosuppressive regimens at the time of the cell collection can significantly impair *ex vivo* manipulation and cell fitness. As a proof-of-concept, we evaluated the robustness of the dual-activation protocol on three patients, one with T1D who underwent pancreatic islet transplantation and two with RA.

Despite the limited sample size, the dual-activation strategy consistently outperformed our standard protocol in boosting the expansion of patient-derived Tregs, while preserving their functionality. The use of cryopreserved PBMCs, combined with the underlying immunosuppressive treatment, represents a challenging and suboptimal starting material. Nevertheless, our protocol allowed the recovery of a high cell yield, suggesting its potential for clinical scalability in patients with compromised Tregs. Further studies involving a larger and more homogeneous cohort of patients will be required to fully validate the clinical utility of this procedure in different autoimmune settings.

Hoffman et al. reported a loss of functionality and FOXP3 expression in CD45RA− Tregs upon multiple restimulations.^17^ In contrast, the second round of stimulation in our protocol enhanced expansion without affecting transduction efficiency, Treg enrichment, or functionality. Also, in terms of memory phenotype, we did not observe major differences between restimulated cells and our standard approach. Speculatively, constitutive FOXP3 expression driven by a strong promoter, together with rapamycin, may better preserve the Treg functional phenotype, as previously reported by Allan et al.^33^

CD45RA+ naive Tregs represent an alternative promising source for the generation of CAR-Tregs, with a potential lower plasticity toward conventional T cells.^6^ Naive Tregs are particularly represented in the thymus, but are rare in the peripheral blood. Their distribution may limit in autologous CAR-Tregs strategy, due to the requirement of extensive sorting procedures with high numbers of starting lymphocytes or of thymuses, usually available only in children undergoing cardiac surgery.^34^^,^^35^

The additional stimulation step required by our protocol might increase the overall complexity of a CAR-Treg strategy. However, CD4+CD25+ Tregs can be used autologously, representing a more immediately translatable approach than naive Tregs, which typically require extensive sorting procedures, access to thymic tissue, or genomic engineering for allogeneic use, resulting in higher costs and greater manufacturing complexity.

Contaminating Tconvs and Treg reprogramming are major challenges in adoptive Treg therapy, especially with the use of anti-self CAR constructs due to the potential risks for healthy tissues. We previously demonstrated how the combination of the *FOXP3* transgene and the culture in the presence of IL-2 and rapamycin can prevent CAR-Treg reprogramming and the contaminant CAR-Tconv outgrowth. Here, we show that this combination is effective in maintaining the optimal safety profile of the cellular product also in the presence of multiple rounds of Treg activation, as demonstrated by the similar phenotype and suppressive capacities of restimulated cells compared with our standard approach and by the absence of killing activity against B cells.

In the literature, no consensus exists about the optimal IL-2 concentration to be used in the Treg manufacturing, thus leading to very heterogeneous approaches. In this study, we compared three different IL-2 concentrations and their impact on the Treg expansion and functionality, without finding significant differences. This likely reflects the intrinsic resilience of Treg to IL-2 due to the constitutive expression of high levels of CD25, the high affinity chain of the IL-2 receptor, as confirmed by several clinical trials evaluating treatments with low-dose IL-2.^36^^,^^37^ We investigated an IL-2 concentration of 250 IU/ml as the lowest amount, although it might be worth exploring the impact of lower quantities, considering its impact on the manufacturing costs, especially in good manufacturing practice (GMP)-grade environments.

Exhaustion is a highly investigated topic for CAR-T cells in cancer, but it is still controversial for the Tregs due to the constitutive expression of the inhibitory receptors on the Treg surface, commonly employed to define an exhausted phenotype. Recently, Lamarche et al. evaluated the role of exhaustion in limiting CAR-Treg functionality, finding the upregulation of specific markers like 4-1BB, LAP, and GARP and a dysfunctional profile in chronically stimulated cells.^18^

To address this, we assessed the effect of the different expansion protocols on the expression of different inhibitory receptors, demonstrating that two rounds of stimulation did not induce exhaustion on CAR-Tregs and did not impair their functionality. The analysis of TOX and TCF expression, two transcription factors associated with the acquisition of exhausted phenotype, revealed no differences between dual-activated and standard CAR-Tregs, further confirming a similar functional profile between the two cellular products.

To confirm the *in vivo* non-inferiority of dual-activated anti-CD19 CAR-Tregs, we established a xeno-GvHD mouse model. The two cellular products showed a similar expansion kinetic when infused in the animals, a comparable control of the circulating human T cell expansion and similar frequencies of infiltrating activated (HLA-DR+) human T lymphocytes in the organs. Collectively, these data confirm the non-inferiority of dual-activated CAR-Tregs compared with our standard product also when employed *in vivo*.

Given their specificity, anti-CD19 CAR-Tregs require the presence of B lymphocytes to become activated and exert their suppressive activity. Considering the predominant T cell role in establishing xeno-GvHD, we decided to interrupt the experiment at 25 days, after the cell injection to avoid confounding effects due to the disappearance of B cells and consequently, the loss of the immunomodulatory CAR-Treg activity. The control of human T cell expansion and the reduced frequency of activated lymphocytes in the organs suggest the presence of an anti-inflammatory CAR-Treg activity and support the comparable functionality between the two products. A longer observational period might help in the future to confirm this aspect.

*Ex vivo* manufacturing requires the use of anti-CD3/CD28 stimulation beads and rapamycin to promote an optimal Treg expansion. Bead removal was confirmed at the end of the culture phase by microscopic inspection and flow cytometry analysis of the side-scatter profile. Rapamycin was removed by cell washing at the end of the expansion phase, as already validated in clinical-grade Treg manufacturing.^4^ These aspects will require a further validation in a future GMP-grade process development.

In conclusion, we propose an optimized CD4+CD25+-derived CAR-Treg expansion protocol based on *FOXP3* transduction and two steps of stimulation in the presence of a low IL-2 concentration and rapamycin. This approach allows to increase the cell expansion up to 4-fold compared with our standard protocol, without impacting the functionality and the safety profile of the cellular product. We are confident that this protocol may help to improve the CAR-Treg manufacturing and scalability to the clinical setting, especially with suboptimal starting material such as patient-derived cells.

## Materials and methods

### Isolation of primary human T cells

PBMCs were collected from healthy donors, after written informed consent, according to the San Raffaele Scientific Institutional Ethical Committee guidelines. PBMCs were isolated by Ficoll density gradient separation (Lymphoprep) and were freshly used for the following protocols of specific T cell isolation. Treg cells were isolated via selection using magnetic cell separation (Miltenyi, CD4+ CD25+ regulatory kit isolation kit human, 130-091-301), according to manufacturer’s instruction. CD4+ CD25− T cells were isolated from the CD25-negative fraction of magnetic cell separation. Isolated cells were then expanded with the following protocols.

### Expansion protocols

Isolated cells were plated at 0.1 × 10^6^ cells/mL in a 24-well plate and stimulated with anti-CD3/anti-CD28 microbeads (Dynabeads) in a 3:1 or 1:1 bead:T cell ratio, in X-VIVO15 (Lonza) supplemented with 10% human serum (HS; Lonza), 1% penicillin-streptomycin (Euroclone), 1% glutamine (Euroclone, 200 mM) and rapamycin (100 nM). After transduction, 500 IU/mL, 1,000 IU/mL, 250 IU/mL of IL-2 (Proleukin) was added and continuously replenished every 2–3 days. After 9 days from activation, cells from double stimulation protocols were collected, counted, and reactivated with anti-CD3/CD28 microbeads at 1:1 ratio adding new beads. Subsequently, restimulated cells were plated at a concentration of 0.15 × 10^6^ cells/mL. On day 14, activation beads were magnetically removed, and cells were counted to assess the expansion rate. Subsequently cells were maintained in culture media supplemented with IL-2 for the subsequent week prior to further phenotypic testing and freezing. Cells were then subsequently thawed and kept in culture for 1–2 days in cytokine-free medium before the functional testing.

### Primary conventional T cell expansion

Conventional CD4+ CD25− T cells (Tconv) were stimulated with anti-CD3/CD28 magnetic beads with 1:3 beads:T cell ratio and supplemented with IL-7 and IL-15 (5 ng/mL, PeproTech). They were cultured in RPMI (Euroclone) supplemented with 10% fetal bovine serum (Euroclone), 1% penicillin-streptomycin, and glutamine. After transduction, IL-7 and IL-15 were continuously replenished every 2 days. On day 6, activation microbeads were magnetically removed, and Tconv cells were counted and were maintained at a concentration of approximately 2 × 10^6^ cells/mL and split as necessary. Cells were then kept in culture up to 14 days in the presence of cytokines for phenotypical testing. Then the cells were frozen and thawed and kept in culture for 1–2 days in a cytokine-free medium before the functional assays.

### Patient-derived Tregs

Frozen PBMCs, obtained from two patients with RA and one with T1D who underwent beta-cell islet transplantation, were thawed and rested overnight. Treg cells were then isolated via selection using magnetic cell separation (Miltenyi, CD4+ CD25+ regulatory kit isolation kit human, 130-091-301), according to the manufacturer’s instruction. Isolated cells were plated at 0.1 × 10^6^ cells/mL in a 96-well flat-bottom plate and stimulated according to the protocol used. The study was approved by the Institutional Ethics Committee of IRCCS Ospedale San Raffaele (approval number: protocollo DRI-MITO 2014. 01/04/2015 and AR_Treg_2020. 12/07/2010) and conducted in accordance with the Declaration of Helsinki.

### Cell lines

B cell precursor cell line (NALM6) was grown in RPMI 1640 (Lonza) supplemented with 10% fetal bovine serum (Euroclone), 1% penicillin/streptomycin (Euroclone), and 1% glutamine (Euroclone). Cells were counted every 3–4 days by trypan blue dye exclusion and plated at a concentration of 0.5–1 × 10^6^ cells/mL.

### Viral vectors

#### CAR constructs

CAR19.28z construct was generated by cloning the antigen-specific single-chain fragment variable (scFv) into a bidirectional LV encoding for the CAR backbone containing an NGFR-derived mutated short spacer (NMS),^38^ a CD28 transmembrane and co-stimulatory domain, and a CD3zeta endodomain under the control of an hPGK promoter. Green fluorescent protein (GFP) was cloned in anti-sense under the control of a mCMV promoter. Fox19CAR LV construct was generated by cloning in a unidirectional LV both the human *FOXP3* gene and an anti-CD19 second-generation CAR under the control of a hPGK promoter. Anti-CD19 CAR was composed of a CD19-specific scFv, a linker peptide derived from the mutated constant portion of IgG4,^39^ and the transmembrane and intracellular portions of the CD28 and the CD3z chains. The two transgenes were linked by a Thosea asigna virus 2 A (T2A) peptide. As control, we generated a unidirectional LV encoding for the *FOXP3* gene under the control of a hPGK promoter.

All employed LV backbones were kindly provided by L. Naldini’s group.

#### Lentiviral vector production

Third-generation replication-defective and self-inactivating LVs were produced by the transient transfection of HEK293T cells. Briefly, a solution containing the packaging plasmid pMDLg/pRRE (containing HIV-1 gag/pol genes); the pILVV01-Rev plasmid (encoding Rev protein); the envelope plasmid encoding the vesicular stomatitis virus glycoprotein, pAdvantage; and the plasmids carrying the transgene of interest was transfected into sub-confluent 293T cells using the calcium chloride precipitation method. Supernatants containing lentiviral particles were collected 48 h later and filtered by a 0.22-μm filter. The supernatant of CAR construct LVs was centrifugated at 20,000 rpm at 20°C for 2h (Beckman Optima XL-100K Ultracentrifuge). Then it was aliquoted and cryopreserved.

#### CD4+CD25+ and CD4+CD25− Treg cell transduction

At day +2 after activation, CD4+25+ and CD4+25− Treg cells were transduced, adding the virus to 1 mL of cells concentrated to 0.2 × 10^6^ cells/mL in X-VIVO15 (Lonza) supplemented with 10% HS (Lonza), 1% penicillin-streptomycin (Euroclone), and glutamine (Euroclone, 200mM) with Rapamycin (100 nM). The LVs were added according to the viral titration to obtain 10 MOI (multiplicity of infection). Treg cells were kept for 24 h at 37°C, and then fresh medium with IL-2 was added in each condition according to the protocol. Transduction efficiency was measured by flow cytometry on day 14 of expansion. Recombinant CD19 reagent (FMC63 PE, Miltenyi) was used for CAR surface staining according to the manufacturer’s instruction.

#### CD4+CD25− T cell transduction

At day +2 after stimulation, activated CD4+CD25− T cells were collected and resuspended in RPMI and treated with 10% fetal bovine serum (Euroclone), 1% penicillin/streptomycin (Euroclone), and 1% glutamine (Euroclone), and 200 μL was plated at a concentration of 2.5 × 10^6^ cells/mL. The LV was added according to the viral titration to achieve an MOI of 10. T cells were kept for 24 h at 37°C, and then fresh medium supplied with IL-7 and IL-15 was added in each condition. Transduction efficiency was measured by flow cytometry on day 14 of expansion, based on the expression of GFP.

### Functional assays

#### Polyclonal suppression assay

Autologous PBMCs (aPBMCs) (target cells) and engineered T reg cells (effector cells) were co-cultured for 6 days at decreasing effector-to-target ratio in the presence of anti-CD3/CD28-coated beads at 1:10 beads:T cell ratio. To detect proliferating cells, PBMCs were stained with the Far Red proliferation dye (Thermo Fisher), while engineered cells were stained with the VioBlue proliferation dye (Thermo Fisher), according to the manufacturer’s instruction. After 6 days, the cells were analyzed by flow cytometry.

Suppression index was calculated as follows:

(Equation 1) $$S.I.=1-\left(\frac{\%\;\text{of}\;\text{proliferating}\;\text{PBMCs}\;\text{in}\;\text{the}\;\text{effector}\;\text{group}}{\%\;\text{of}\;\text{proliferating}\;\text{PBMCs}\;\text{alone}}\right)$$

#### Antigen-specific suppression assay

CD19+ B lymphocytes (target cells) were negatively sorted from cryopreserved PBMCs using the B cell isolation kit II (Miltenyi), according to the manufacturer’s instruction. Sorted B cells were labeled with the Far Red proliferation dye (Thermo Fisher), while effector cells (CAR-Treg) were stained with the VioBlue proliferation dye (Thermo Fisher) according to the manufacturer instructions. Labeled cells were co-cultured at decreasing effector-to-target ratio. Cells were analyzed by flow cytometry. B cells were stimulated with 0.5 μg/mL of CD40L (TNFSF5 Bio-Techne), IL-21 (100 ng/mL), and 20 μg/mL of AffiPure F(ab’)_2_ Fragment Goat Anti-Human IgA + IgG + IgM (H + L) (Jackson Immunoresearch Laboratories). After 3 days, the cells were stained for CD80 and CD86 and analyzed by flow cytometry.

Suppression index was calculated as follows:

(Equation 2) $$S.I.=1-\left(\frac{\%\text{proliferating}\;B\;\text{cells}\;\text{when}\;\text{cultured}\;w\text{ith}\;\text{Tregs}}{\%B\;\text{cell}s’\;\text{proliferation}\;\text{alone}}\right)$$

#### Killing assay

B cell precursor cell line (NALM6) or primary B cells (target cells), and engineered T/Treg cells (effector cells) were co-cultured at decreasing effector-to-target ratios. To detect proliferating cells, NALM6 or B cells were stained with the Far Red proliferation dye, while engineered cells were stained with the VioBlue proliferation dye, according to the manufacturer’s instruction. After 3 days, the cells were analyzed by flow cytometry, and the elimination index was calculated as follows:

(Equation 3) $$E.I.=1-\left(\frac{\text{number}\;\text{of}\;\text{target}\;\text{cells}\;\text{in}\;\text{presence}\;\text{of}\;\text{redirected}\;T/\text{Treg}\;\text{cells}}{\text{number}\;\text{of}\;\text{viable}\;\text{target}\;\text{cells}\;\text{in}\;\text{presence}\;\text{of}\;\text{control}\;T/\text{Treg}\;\text{cells}}\right)$$

#### Multiple lymphocyte reaction

aPBMCs were stimulated with anti-CD3/CD28 Dynabeads (Thermo Fisher Scientific) at a ratio of 2:1 (bead:T cell ratiol)*.* Simultaneously CAR-Tregs or untransduced Tregs were stained with the VioBlue proliferation dye (Thermo Fisher) according to the manufacturer’s instruction and plated at decreasing ratios in the presence of irradiated (60 Gy) alloreactive PBMCs. After 24 h, the microbeads were magnetically removed from aPMBCs and the cells were stained with Far Red (Thermo Fischer) and plated on top of the previously plated Tregs. After 5 days of co-culture, cells were analyzed by flow cytometry, and the suppression index was calculated as previously reported.

#### IL-6 challenge assay

Alloreactive PBMCs (target) and CAR-Treg cells (effector) were stimulated with anti-CD3/CD28 microbeads at 1:10 beads:T cell ratio and co-cultured at decreasing effector-to-target ratios in the presence of the beads. To monitor proliferation, allo-PBMCs were stained with the Far Red proliferation dye (Thermo Fisher) and CAR-Treg cells were stained with the VioBlue proliferation dye (Thermo Fisher), according to the manufacturer’s instruction. Interleukin-6 (IL-6) was added to the co-culture at 25 ng/mL, and the effect was compared with a parallel co-culture performed under identical conditions but in the absence of IL-6. CD4+CD25+ Tregs cultured with our standard protocol and without rapamycin were used as controls. After 6 days of co-culture, cells were analyzed by flow cytometry, and the suppression index was calculated as previously reported.

#### Retrieval of integration sites (ISs) from cell DNA

Genomic DNA was extracted using the QIAamp DNA Blood Mini Kit (QIAGEN) or QIAamp DNA Micro Kit (QIAGEN), based on a cell pellet. For LV-transduced cells, the number of vector copies per genome were determined by quantitative droplet-digital PCR. The LV psi (φ) vector sequence were analyzed on 20 ng of total genomic DNA and normalized to a region of the human *GAPDH* gene. For the retrieval of vector ISs from genomic DNA, we adopted a sonication-based linker-mediated (SLiM) PCR method.^40^ Briefly, genomic DNA was sheared using a Covaris E220 ultrasonicator, generating fragments with an average size of 1,000 bp. The fragmented DNA was then split into three technical replicates and subjected to end repair and 3′ adenylation using the NEBNext Ultra DNA Library Prep Kit for Illumina (New England Biolabs) and then ligated (DNA Technologies ligation kit) to an LC with known sequence. Ligation products were then subjected to 35 cycles of exponential PCR with primers complementary to the LV long terminal repeat (LTR) and the linker cassette (LC). Next, 10 additional PCR cycles were done to add the sequences required for sequencing and a second 8-nucleotide DNA barcode. Finally, the amplification products were sequenced using the Illumina NextSeq platform (Illumina).

#### Bioinformatics analysis

Sequencing data were processed using the VISPA2 bioinformatics pipeline (https://github.com/giuliospinozzi/vispa2),^41^ which extracts genomic regions adjacent to the vector LTR and aligns them to the human reference genome. In summary, paired end reads undergo quality filtering, barcode recognition for sample demultiplexing, and mapping of the genomic portion to the GRCh38/hg38 assembly, after which each uniquely aligned integration site is annotated with its closest RefSeq gene. VISPA2 filters out: (1) reads lacking the full LV LTR immediately downstream of the amplification oligonucleotide (12 nucleotides [nt]), (2) reads shorter than 19 nt, (3) reads that fail to align to the reference genome, (4) reads mapping to more than one genomic location, (5) reads whose genomic alignment exceeds 1.2 kb, and (6) read pairs mapping to different chromosomes or to opposite strands of the same chromosome. Integration site quantification and downstream analysis were performed using the ISAnalytics package (https://doi.org/10.18129/B9.bioc.ISAnalytics).^42^ ISAnalytics implements the sonicLength fragment-based strategy to estimate the abundance of each integration site and calculates the Shannon diversity index (H-index) as a measure of clonal diversity.

#### *In vivo* humanized model of xeno-GvHD

The experimental protocol was approved by the Institutional Animal Care and Use Committee (IACUC) with the number 1359. Seven- and 8-week-old NSG mice (NSG, JAX 005557, RRID IMSR_JAX: 005557, Charles-River Italia) were intravenously infused with 5 × 10^6^ healthy donor PBMCs. After 1 week, PBMC-injected NSG mice were divided into 4 groups of treatment: 3.5 × 10^6^ autologous CAR-Tregs expanded with the standard protocol (STD), 3.5 × 10^6^ autologous CAR-Tregs expanded with the double activation protocol (DS), 5 × 10^6^ autologous untransduced Tregs (UT), or PBS as control (CTRL). Mice were clinically monitored and weighed twice a week. Prior to cell treatment and +3, +7, +11, +18, and +25 days post-cell injection peripheral blood was collected for PBMC and Tregs engraftment analysis. Thirty days after cell injection, mice were euthanized, and the spleen and bone marrow were harvested for flow cytometry evaluations.

#### Flow cytometry panels

For Treg phenotype, cells were labeled with titrated fluorescent monoclonal antibodies specific for antiCD19 CAR (FMC63, Miltenyi), CD3 (BioLegend), CD4 (Miltenyi), FOXP3 (eBioscience), CD45RA (BD Biosciences), CD62L (BD Biosciences), TIGIT (BioLegend), LAP (eBioscience), Helios (BioLegend), CD25 (Miltenyi), CD27 (BD Biosciences), CD137 (BioLegend), CD127 (Miltenyi), GARP (BD Biosciences), CTLA-4 (BioLegend), ICOS (BD Biosciences), and GITR (BioLegend).

For Treg exhaustion phenotype, cells were labeled with titrated fluorescent monoclonal antibodies specific for LAG3 (Miltenyi), CD137 (BioLegend), CD25 (BioLegend), CD4 (BioLegend/Invitrogen OKT4), HLA-DR (BD Horizon), CTLA4 (BioLegend), PD1 (BioLegend), KLRG1 (BioLegend), CD3 (BD Horizon), CD45RA (BD Biosciences), CD62L (BD Biosciences), TIM3 (Miltenyi), 2B4 (BioLegend), TIGIT (Invitrogen), TOX (Thermo Fisher), and TCF (BioLegend).

For intra-nuclear staining, lymphocytes were first stained with surface antibodies and then washed, fixed, and permeabilized with FOXP3 staining buffer set (Miltenyi), according to manufacturer’s instruction. Intracellular staining was performed with anti-Helios and anti-FOXP3 antibodies.

### Statistical analysis

Statistical analyses were performed with Prism 10 (GraphPad Software). Normal distribution of continuous variables was assessed using the Shapiro-Wilk test. One way or two-way ANOVA test was used when comparing three or more different groups, and Tukey test was employed for the post hoc analysis. For all comparisons, a *p* value <0.05 was considered statistically significant.

## Data availability

Data are available upon request to the corresponding authors.

## Acknowledgments

This work was supported by the Italian Ministry of Research and University (PRIN 2017WC8499), Italian Ministry of Health and Alliance Against Cancer (Ricerca Corrente CAR T project: RCR-2019-23669115), and the EU IMI initiative (T2EVOLVE consortium) to B.B. This work was partially supported by the European Union - Next Generation EU, Mission 4, Component 1 CUP D46F23000070004 to A.U.

## Author contributions

A.U. and A.M. contributed to conceptualization, formal analysis, investigation, methodology, visualization, and writing – original draft. C.B.-B., B.C., P.C., S.C., E.B., L.R., and D.C. contributed to investigation and formal analysis. G.A.R., P.M., and L.D. contributed to patient selection. P.M., R.G., and F.C. contributed to conceptualization and writing – review and editing. C.B. and M.D. contributed to conceptualization, methodology, project administration, resources, supervision, and writing – review and editing.

## Declaration of interests

C.B. and F.C. are inventors on different patents on cancer immunotherapy and genetic engineering. C.B. has been a member of the Advisory Board and a consultant for Molmed, Intellia, TxCell, Novartis, GSK, Allogene, Kite/Gilead, Miltenyi, Kiadis, Evir, and Janssen and received research support from Molmed s.p.a and Intellia Therapeutics. R.G. discloses speaking honoraria from Biotest, Pfizer, BMS, MSD, Medac, Neovii, Kyverna, and Magenta. A.U., C.B.-B., P.C., M.D., and C.B. have been collaborating with GentiBio.

## Supplemental information

Document S1. Figures S1–S10

Document S2. Article plus supplemental information
