# Supplementary material for: Double activation and low IL-2 manufacturing protocol increase expansion of healthy and patient-derived CAR-Tregs
Source: Mol Ther Adv. 2026 Jul 9;34(3):201808. doi: 10.1016/j.omta.2026.201808 (PMC13449783; doi:10.1016/j.omta.2026.201808)
Supplement: Supplementary file 1 [file mmc1.pdf]

OMTA, Volume *34*

Supplemental information

Double activation and low IL-2 manufacturing protocol increase expansion of healthy and patient-derived CAR-Tregs

Alessia Ugolini, Anna Miceli, Clara Bercher-Brayer, Susanna Cesarano, Barbara Camisa, Pierluigi Carulli, Paolo Monti, Giuseppe Alvise Ramirez, Elisa Bonomi, Laura Rudilosso, Lorenzo Dagna, Daniela Cesana, Raffaella Greco, Fabio Ciceri, Chiara Bonini, and Matteo Doglio

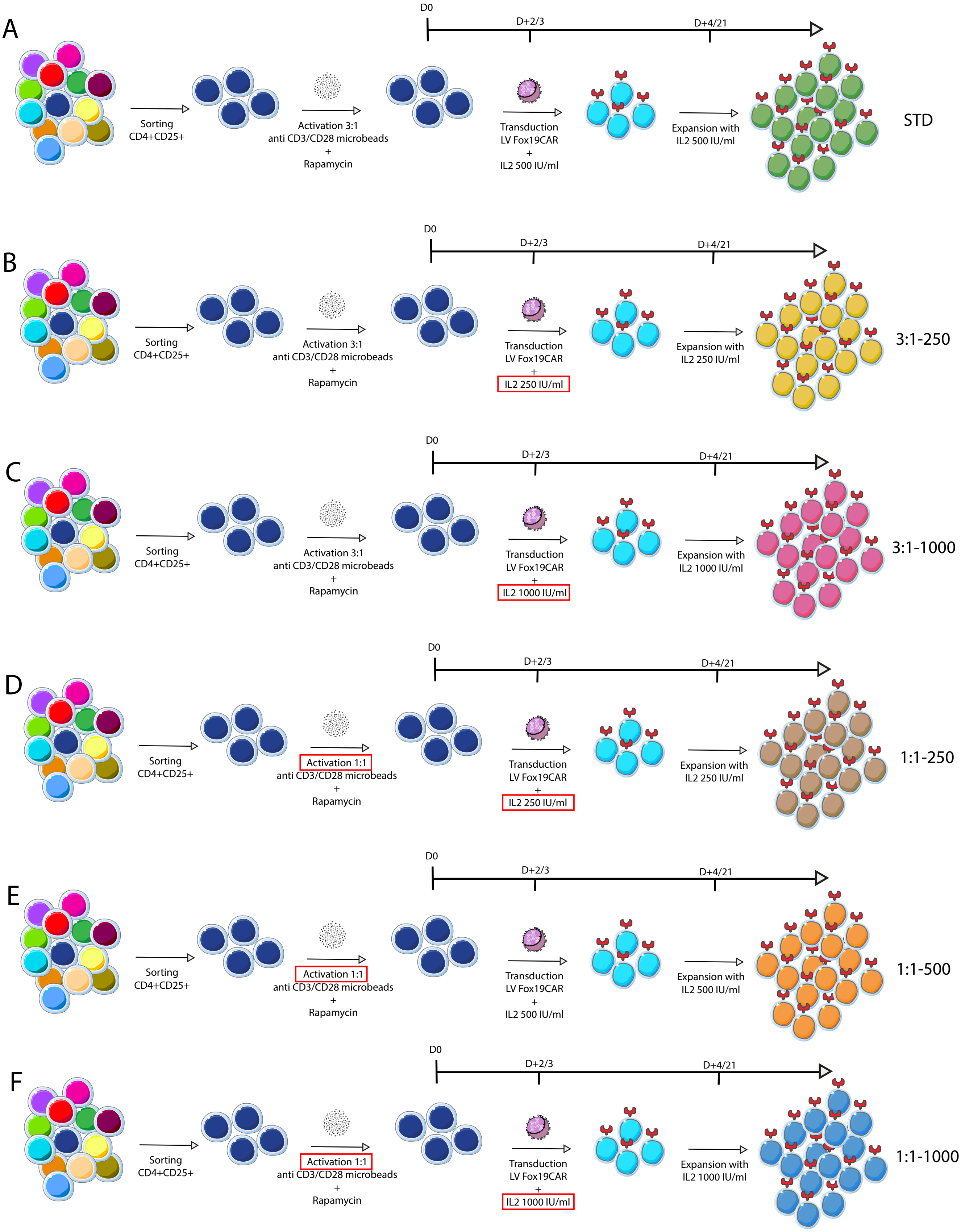

A
D0
D+2/3
D+4/21
Sorting
CD4+CD25+
Activation 3:1
anti CD3/CD28 microbeads
+
Rapamycin
Transduction
LV Fox19CAR
+
IL2 500 IU/ml
Expansion with
IL2 500 IU/ml
STD
B
Activation 3:1
anti CD3/CD28 microbeads
+
Rapamycin
Transduction
LV Fox19CAR
+
IL2 250 IU/ml
Expansion with
IL2 250 IU/ml
3:1-250
C
Activation 3:1
anti CD3/CD28 microbeads
+
Rapamycin
Transduction
LV Fox19CAR
+
IL2 1000 IU/ml
Expansion with
IL2 1000 IU/ml
3:1-1000
D
Activation 1:1
anti CD3/CD28 microbeads
+
Rapamycin
Transduction
LV Fox19CAR
+
IL2 250 IU/ml
Expansion with
IL2 250 IU/ml
1:1-250
E
Activation 1:1
anti CD3/CD28 microbeads
+
Rapamycin
Transduction
LV Fox19CAR
+
IL2 500 IU/ml
Expansion with
IL2 500 IU/ml
1:1-500
F
Activation 1:1
anti CD3/CD28 microbeads
+
Rapamycin
Transduction
LV Fox19CAR
+
IL2 1000 IU/ml
Expansion with
IL2 1000 IU/ml
1:1-1000

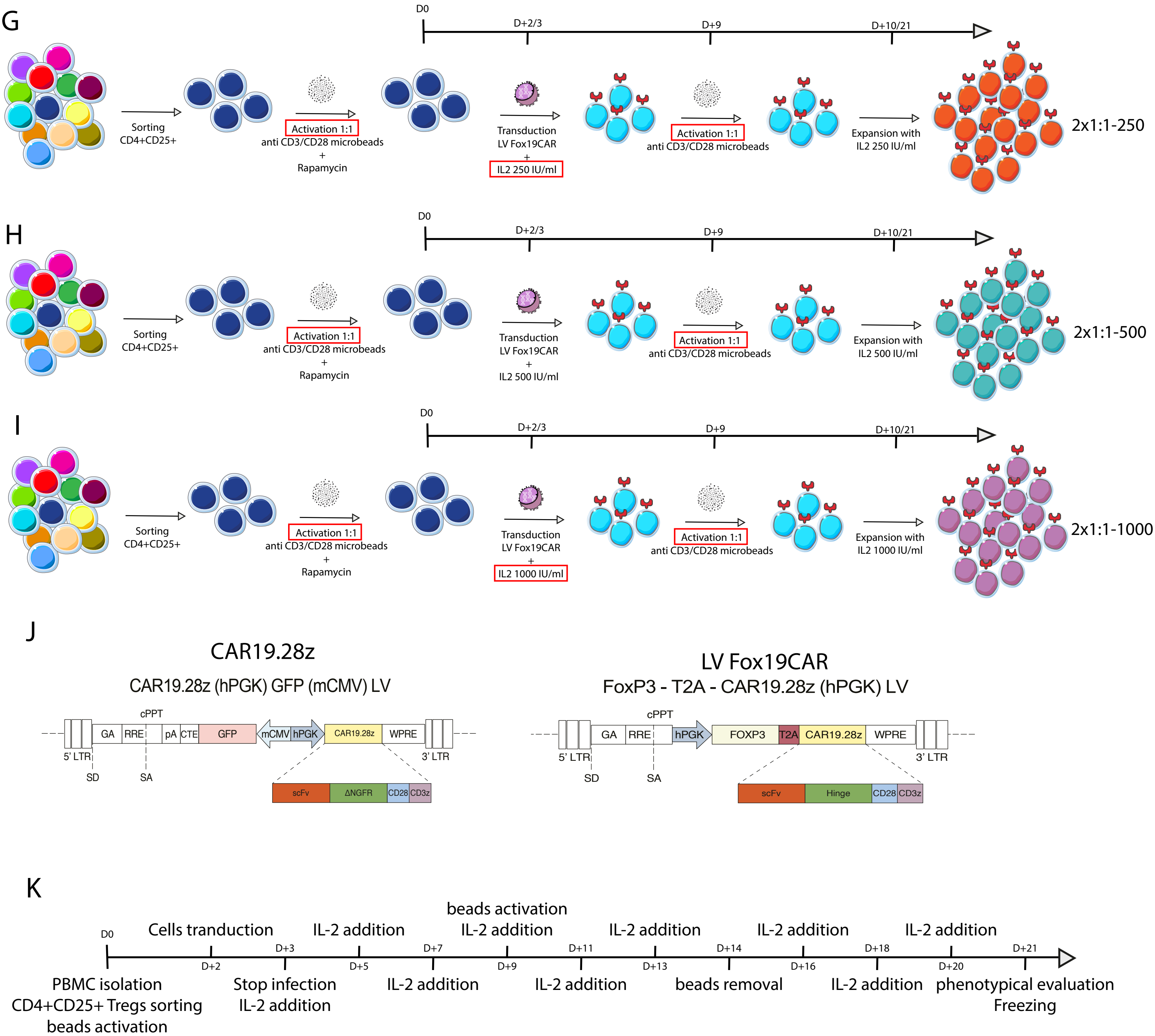

Fig. S1. Schematic representation of the tested expansion protocols, timeline and vectors used.
A-I. PBMC from healthy donors have been isolated and CD4+CD25+ were subsequently magnetically sorted and stimulated following the respective protocol. The standard protocol (STD) was based on 1 round of Treg activation with CD3/CD28 beads at a 3:1 beads-to-T cell ratio and IL-2 at 500UI/ml. New protocols used a 3:1 or 1:1 beads-to-T cell ratios with IL-2 at 250, 500 or 1000 IU/ml. Protocols involving 2 rounds of Treg activation at 1:1 beads-to-T cell ratio (2x1:1) with IL-2 at 250, 500 or 1000 UI/ml were also tested. All steps differing from the standard protocol are highlighted in a red square J. Schematic representations of the vectors used. A bi-directional Lentiviral Vector (CAR19.28z), encoding for and anti-CD19 second generation CAR under the control of the PGK promoter, and the Green-Fluorescent Protein (GFP) controlled by the mCMV promoter. An unidirectional Lentiviral Vector (Fox19CAR) encoding for the FoxP3 gene and an anti-CD19 second-generation CAR under the control of a PGK promoter. K. Timeline showing the main steps in cell manipulation.

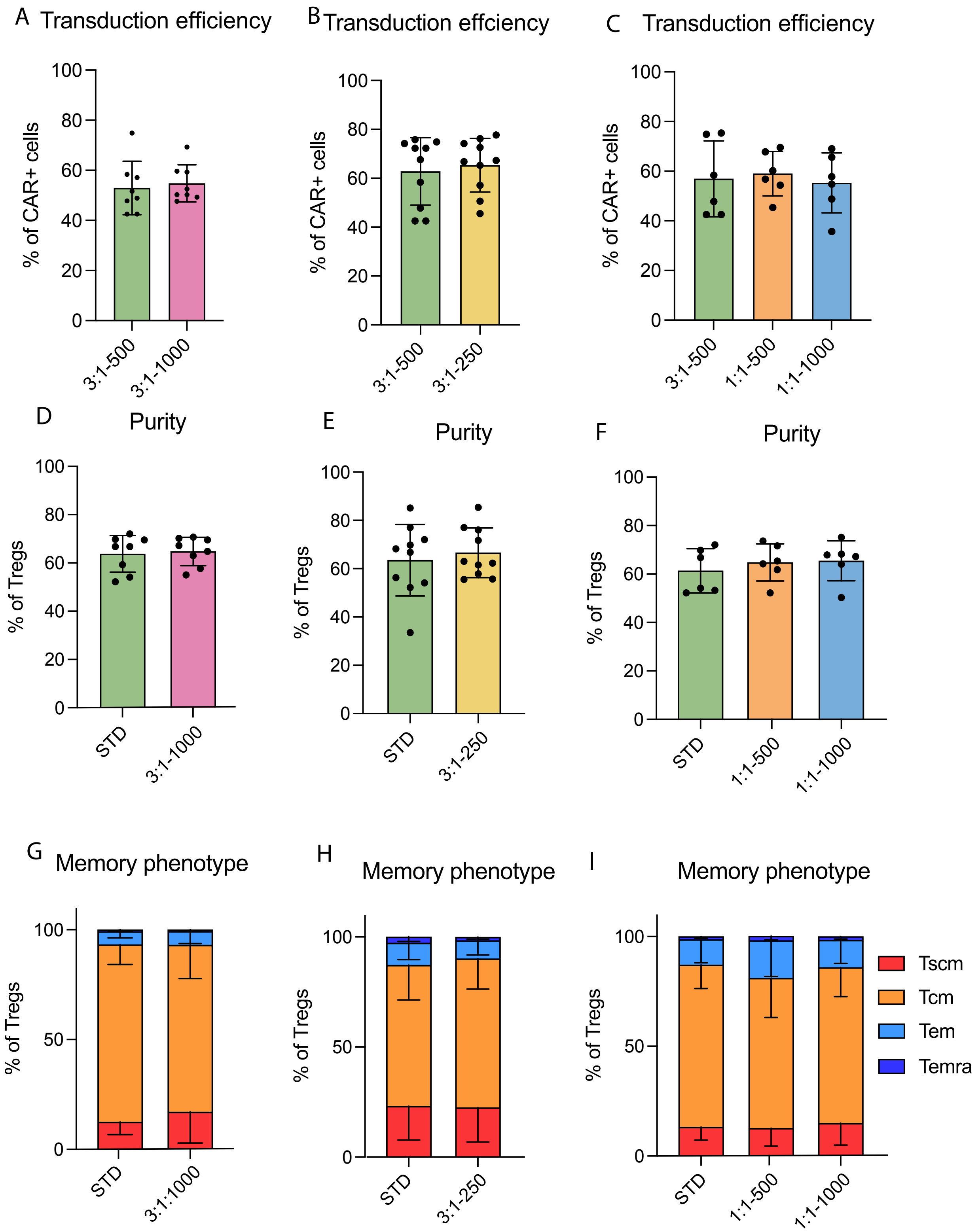

Fig. S2. Transduction efficiency, purity and memory phenotype of 3:1-250/1000 and 1:1-500/1000 protocols.
A-C. Transduction efficiency of Fox19CAR LV on Tregs assessed by flow cytometry at day +14. CAR+ cells were identified with CD19 CAR FMC63 Idiotype Antibody. D-F. Percentage of regulatory T cells among T lymphocytes at day +21 defined as CD3+CD4+CD25+CD127-FOXP3+ cells.G-I. Percentage of memory subsets detected by flow cytometry at day +21, identified as follows: CD62L+CD45RA+ memory stem T cells (Tscm), CD62L+CD45RA- central memory T cells (Tcm), CD62L-CD45RA- effector memory T cells (Tem) and CD62L-CD45RA+ effector memory T cells CD45RA+ (Temra). All data are represented as Mean ± SD.

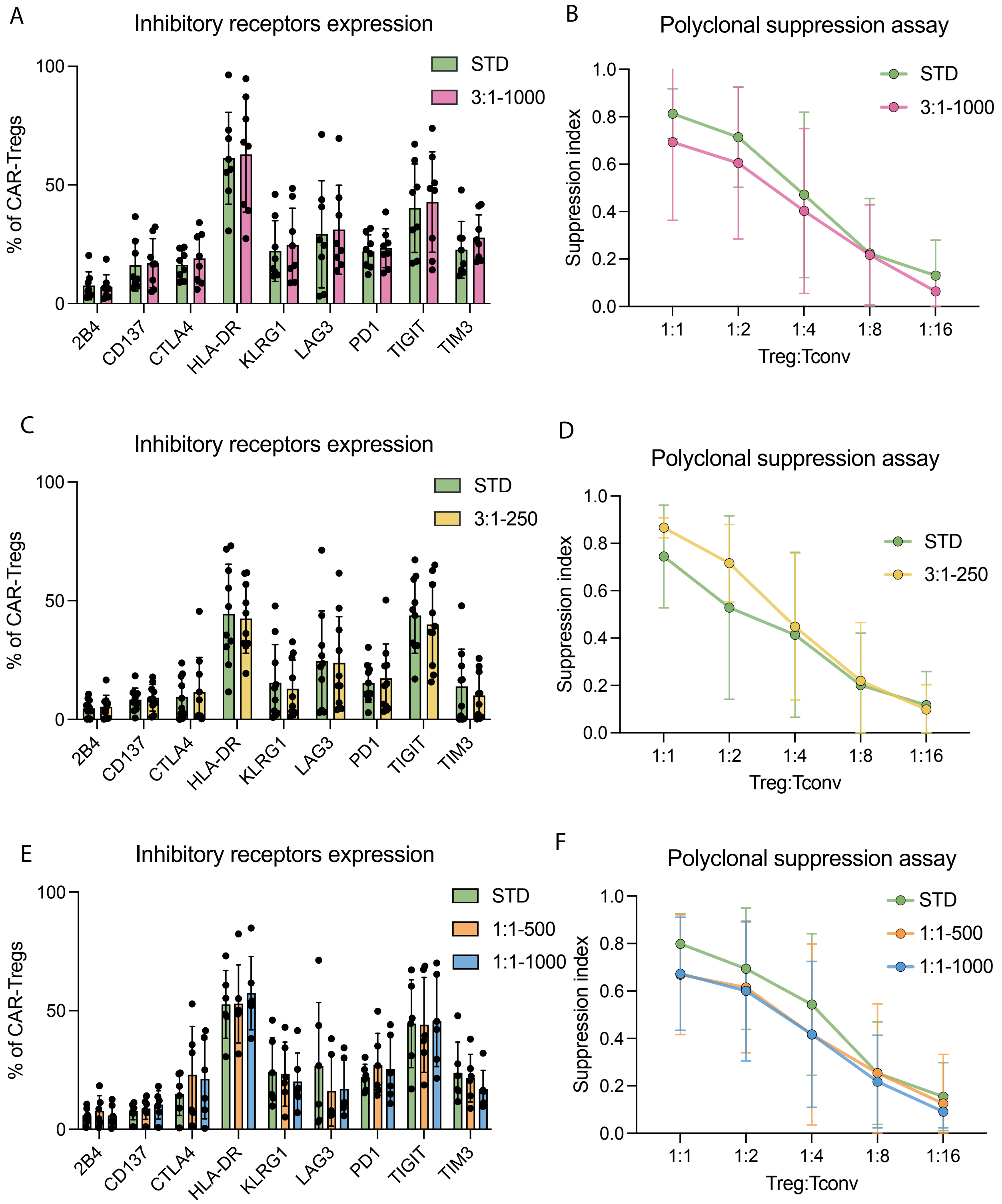

Fig. S3. Exhaustion markers expression and polyclonal suppressive abilities of 3:1-250/1000 and 1:1-500/1000 protocols. A, C, E. Percentage of CAR-Treg (CD3+CD4+CD25+FOXP3+CAR+) cells expressing activation and exhaustion markers detected by flow cytometry after 21 days of culture. B, D, F. Polyclonal suppressive capacities of CAR-Tregs at different Treg : Tconv ratios. Results are expressed in terms of Suppression Index. All data are represented as Mean ± SD.

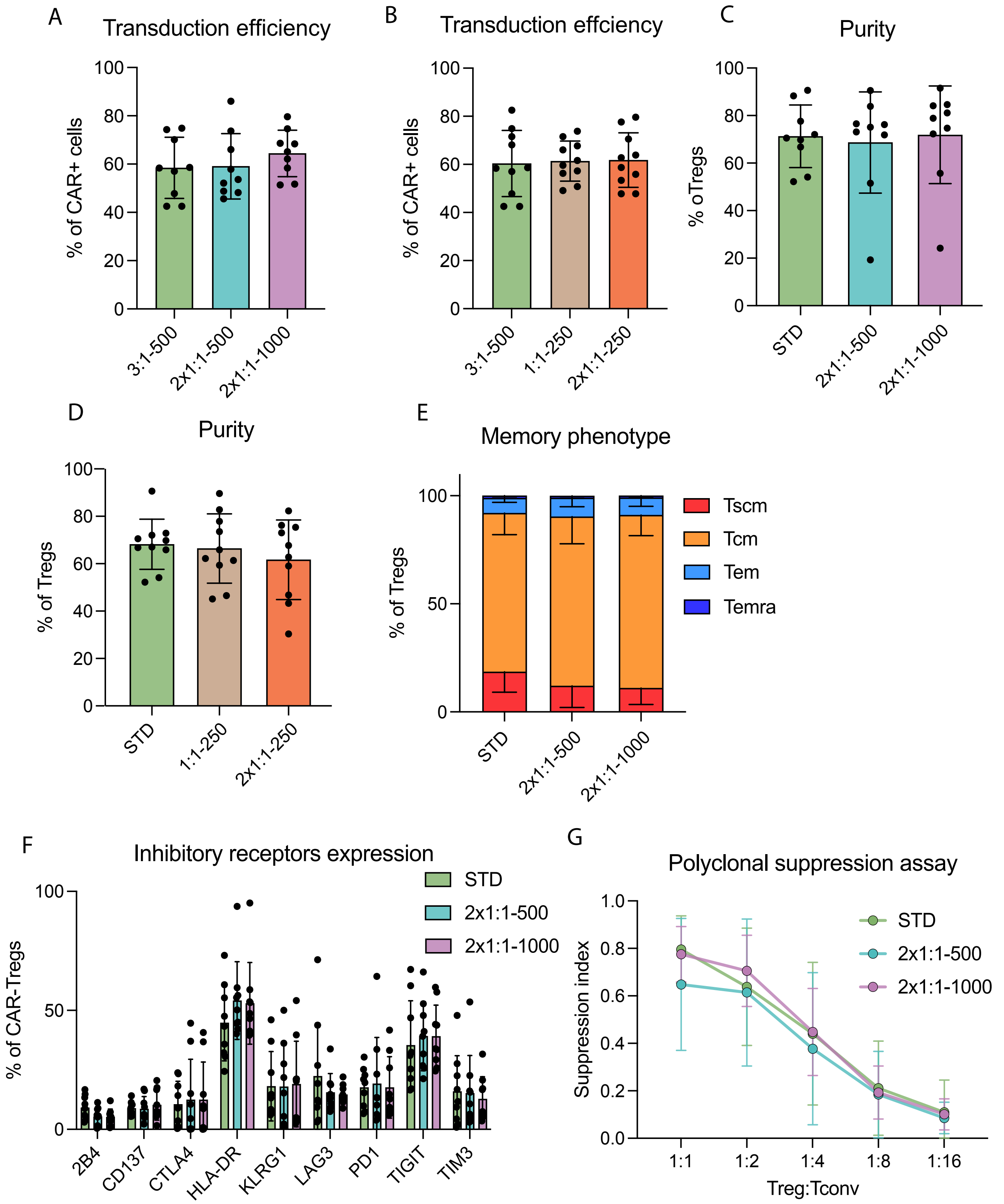

Fig. S4. Phenotypical and functional characterization of 2x1:1-500 and 2x1:1-1000 protocols.
A, B. Transduction efficiency of Fox19CAR LV on Tregs assessed by flow cytometry at day +14. CAR+ cells were identified with CD19 CAR FMC63 Idiotype Antibody. C, D. Percentage of regulatory T cells among T lymphocytes at day +21 defined as CD3+CD4+CD25+CD127-FOXP3+ cells. E. Percentage of memory subsets detected by flow cytometry at day +21, identified as follows: CD62L+CD45RA+ memory stem T cells (Tscm), CD62L+CD45RA- central memory T cells (Tcm), CD62L-CD45RA- effector memory T cells (Tem) and CD62L-CD45RA+ effector memory T cells CD45RA+ (Temra). F. Percentage of CAR-Treg (CD3+CD4+CD25+FOXP3+CAR+) cells expressing activation and exhaustion markers detected by flow cytometry after 21 days of culture. G. Polyclonal suppressive capacities of CAR-Tregs at different Treg : Tconv ratios.Results are expressed in terms of Suppression Index. All data are represented as Mean ± SD.

A

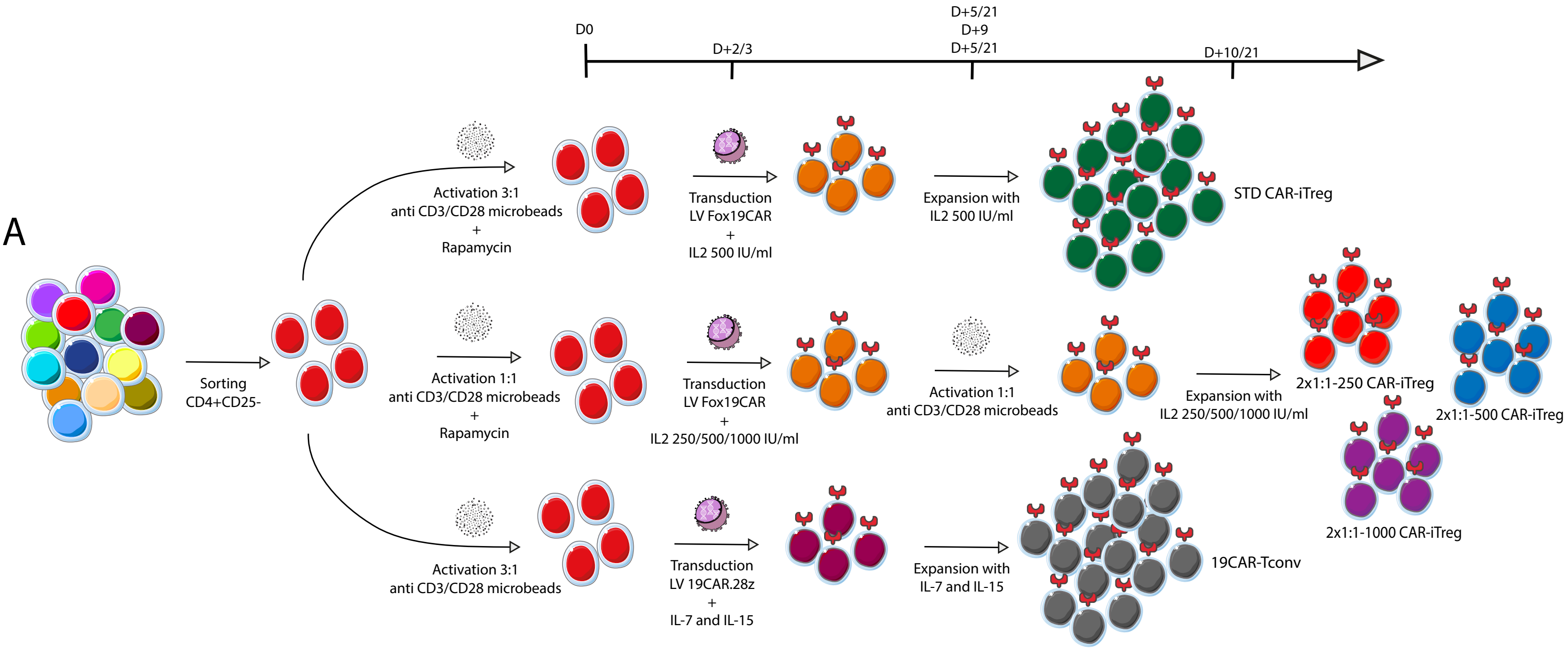

Fig. S5. Schematic representation of CD4+CD25- cells. (A) CD4+CD25- cells were isolated as the negative fraction of the CD4+CD25+ Treg sorting. Cells have been then transduced either with LV 19FoxCAR vector and expanded in presence of rapamycin and IL-2 with different expansion protocols (STD, 2x1:1-250, 2x1:1-500, and 2x1:1-1000), or transduced with LV CAR19.28z and expanded with IL-7 and IL-15 for the generation of the cytotoxic counterpart (19CAR-Tconv).

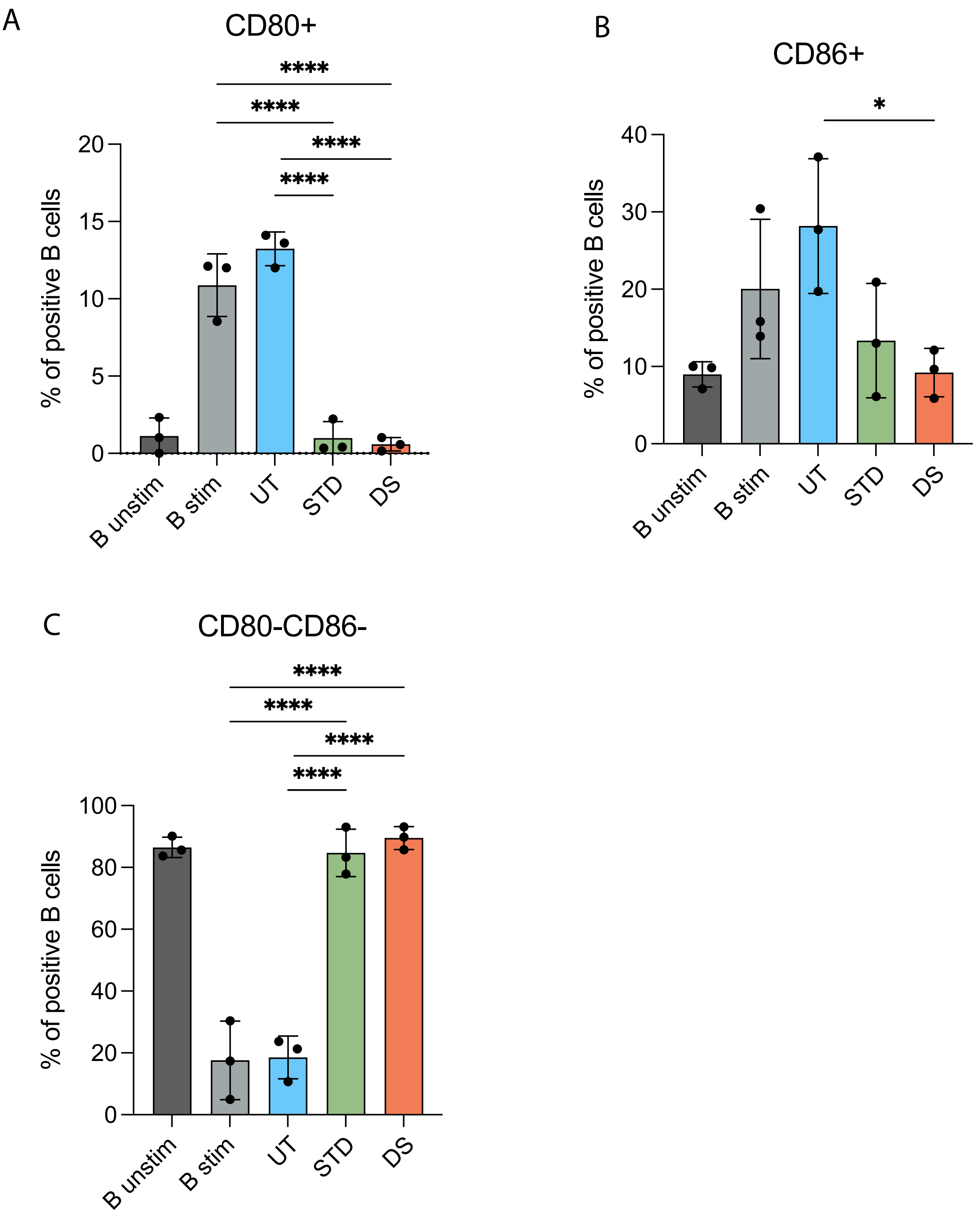

Fig. S6. CD80/CD86 expression of B cells.
A. Percentage of CD80+CD86neg B cells after 72h coculture with Tregs or left alone. Two-way ANOVA with Tukey correction for multiple comparison ****p-value <0.0001. B. Percentage of CD86+CD80neg B cells after 72h coculture with Tregs or left alone. Two-way ANOVA with Tukey correction for multiple comparison *p-value <0.0382. C. Percentage of CD80+CD86neg B cells after 72h coculture with Tregs or left alone. Two-way ANOVA with Tukey correction for multiple comparison ****p-value <0.0001. All data are represented as Mean ± SD.

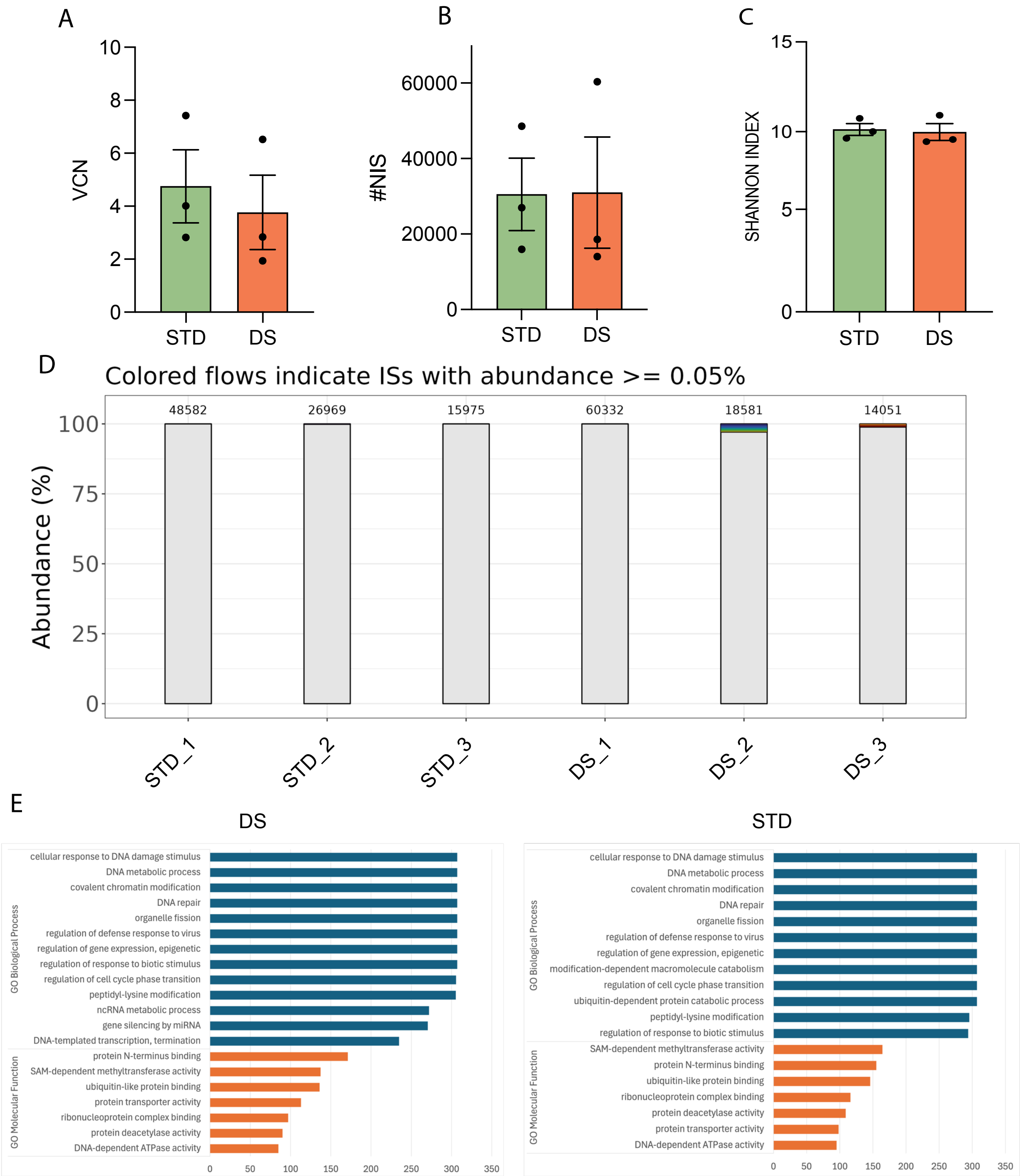

Fig. S7. Lentiviral engineering and dual-activation safety validation.
A. Mean ± SEM of Vector Copy Number (VCN) values for STD versus DS, with individual healthy donors (HD) values overlaid. B. Number of uniq integration sites (#IS) for STD versus DS, shown as mean ± SEM with individual values. C. Clonality of integration sites (ISs) measured by the Shannon diversity index (H-index), shown as mean ± SEM with individual values. D. Alluvial plot showing the distribution of integration sites (IS) in HD 1, 2, 3 under STD and DS conditions. Colored flows highlight those IS that reach an abundance of at least 0.05% in at least one sample, whereas all IS that remain below this threshold are aggregated into a single background layer. Numbers above each bar indicate the total count of unique integration sites identified in that sample. E. Gene Ontology (GO) enrichment analysis of integration sites reported for DS condition (on the left) and STD (on the right) condition. For all comparisons (VCN, number of integration sites, and Shannon diversity), statistical testing between STD and DS conditions was performed using the Mann–Whitney test. All data are represented as Mean ± SD.

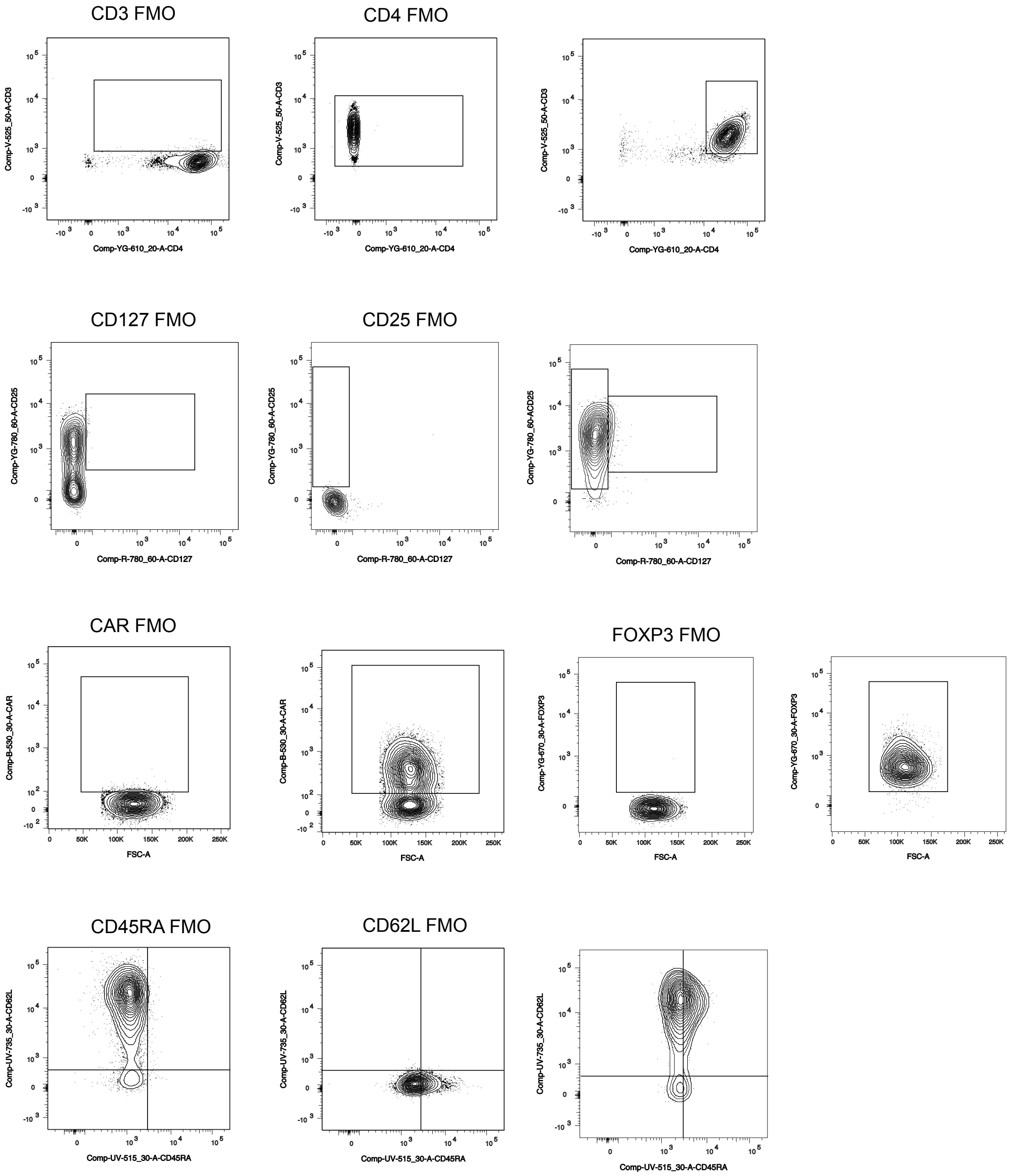

Fig. S8. Fluorescence Minus One (FMO) of the expression markers for Treg purity and memory phenotypes.

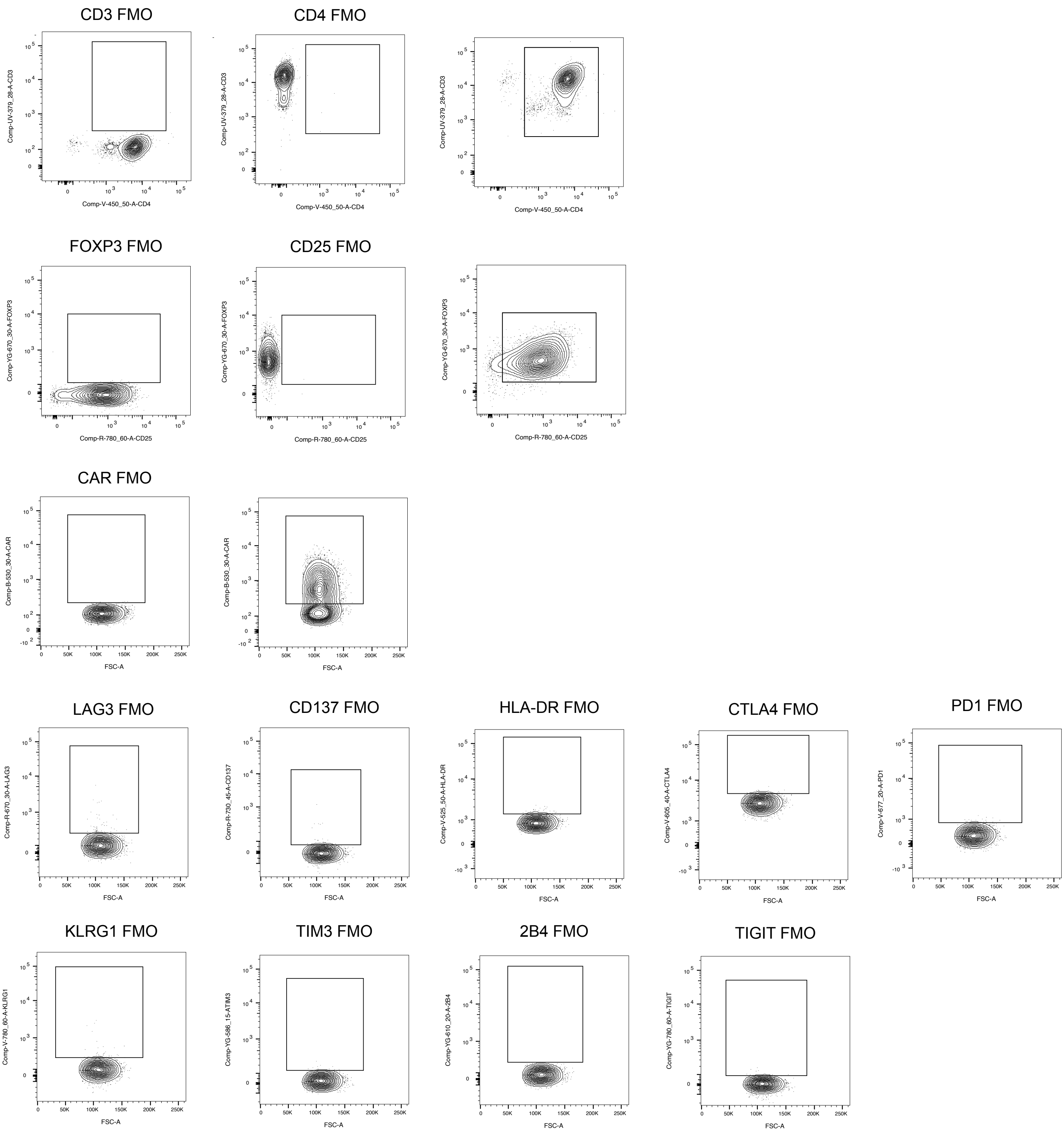

Fig. S9. Fluorescence Minus One (FMO) of the expression markers for Treg exhaustion phenotypes.

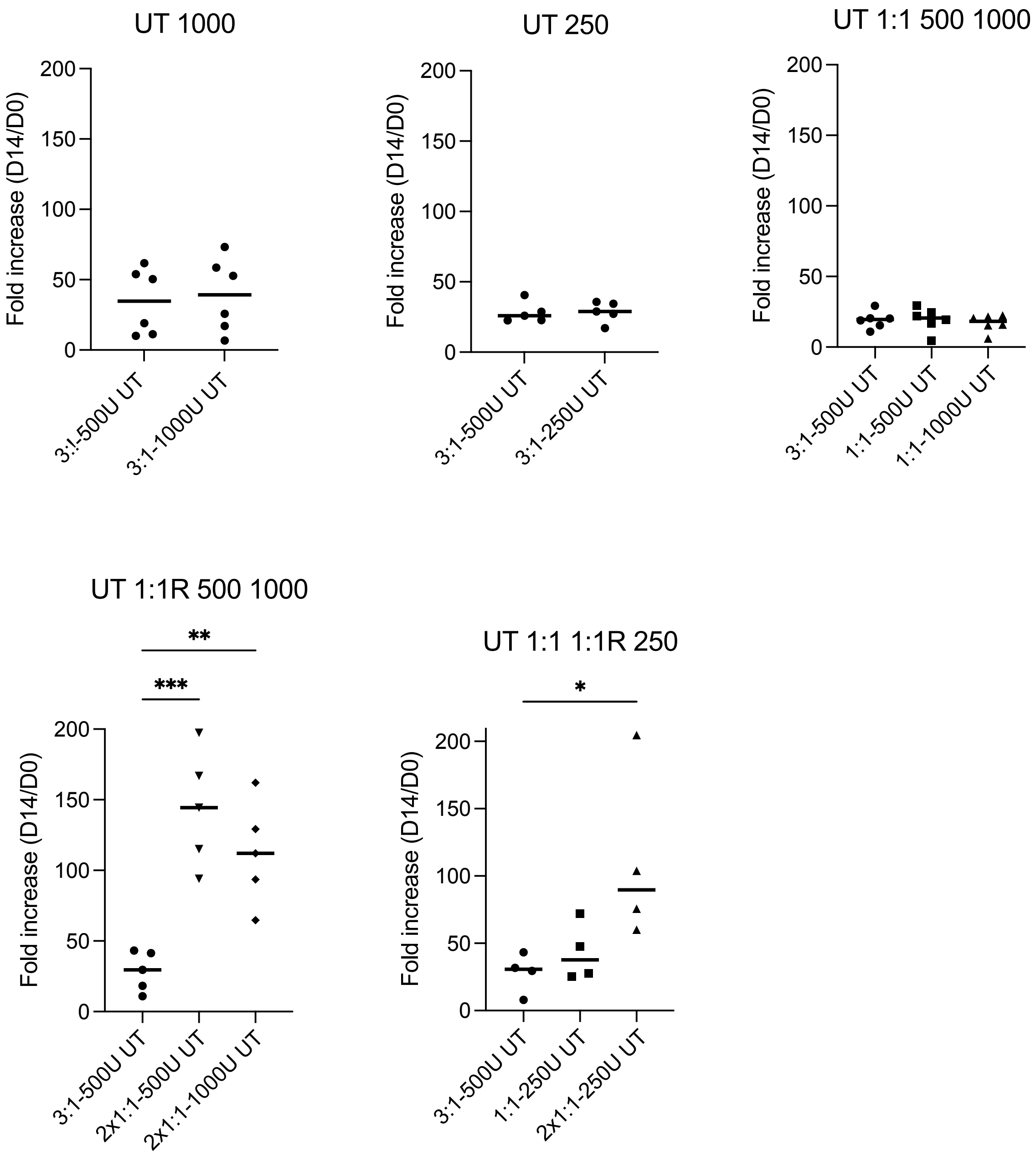

Fig. S10. Expansion rate of untransduced Tregs (UT) cultured with different expansion protocols.
Fold increase was calculated dividing the number of cells obtained at day +14 with the one at day 0. Two-way ANOVA with Tukey correction for multiple comparison ***p-value <0.0044 2x1:1-1000 vs. STD; **p-value<0.0003 2x1:1-500 vs. STD; *p-value <0.0424 2X1:1-250 vs. STD. All data are represented as Mean ± SD.
